# SDK1 as an Independent Prognostic Biomarker in Primary Glioma: A Multi-Cohort Validation Study with Functional Characterization

**DOI:** 10.3390/ijms27104199

**Published:** 2026-05-08

**Authors:** Jun Hyun Lee

**Affiliations:** Seoul National University Hospital, Seoul 03080, Republic of Korea; gamie7@snu.ac.kr

**Keywords:** SDK1, glioma, prognosis, tumor microenvironment, extracellular matrix, immune checkpoint, biomarker, mesenchymal transition, tumor mutational burden

## Abstract

Glioma prognosis is shaped by molecular markers such as IDH mutation, WHO grade, and MGMT methylation, yet heterogeneity persists within defined subgroups. Sidekick Cell Adhesion Molecule 1 (SDK1), an immunoglobulin superfamily member mediating homophilic adhesion, has been documented in glioma tissue but lacks systematic prognostic evaluation. I assessed SDK1’s prognostic value using the Chinese Glioma Genome Atlas (CGGA, N = 503) and The Cancer Genome Atlas (TCGA, N = 572) through multivariate Cox regression, subgroup analyses, differential gene expression, pathway enrichment, ssGSEA-based immune profiling, and molecular subtype association. High SDK1 expression was independently associated with poor overall survival in both cohorts (CGGA: adjusted HR = 1.48, 95% CI 1.16–1.89, *p* = 0.002; TCGA: HR = 1.76, 95% CI 1.19–2.61, *p* = 0.005; pooled HR = 1.55, I^2^ = 0%). Effect estimates varied across subgroups, with significant associations in WHO grade IV and IDH-wildtype strata but not in grade II or older patients. Cross-validated differentially expressed genes were enriched in extracellular matrix organization and focal adhesion pathways. Notably, SDK1 expression showed weak but statistically significant correlations with COL1A1-associated mesenchymal program scores (CGGA: R = 0.12, *p* = 0.008; TCGA: R = 0.15, *p* < 0.001) and oncostream-related gene signatures (CGGA: R = 0.16, *p* < 0.001; TCGA: R = 0.086, *p* = 0.039), suggesting a modest association with mesenchymal invasion programs. SDK1-high tumors showed elevated M2 macrophage and Treg signatures with upregulated immune checkpoints, though cohort-dependent differences were observed. Multivariate Cox analysis demonstrated that the prognostic significance of SDK1 is independent of tumor mutational burden (TMB), with no significant correlation or interaction observed between them (*p* > 0.05). SDK1 is a candidate prognostic biomarker in glioma co-occurring with ECM remodeling and immunosuppressive features, warranting experimental validation for clinical translation.

## 1. Introduction

Glioma accounts for approximately 80% of malignant primary brain tumors and remains among the most challenging cancers to treat [1]. Despite advances in surgical techniques, radiotherapy, and temozolomide-based chemotherapy [2,3], prognosis remains poor, with glioblastoma (WHO grade IV) median overall survival of approximately 14–16 months. Landmark genomic studies of glioblastoma identified key driver alterations [4], and the 2016 and 2021 WHO classifications of central nervous system tumors have integrated molecular markers—including isocitrate dehydrogenase (IDH) mutation status [5]; 1p/19q codeletion; and O6-methylguanine-DNA methyltransferase (MGMT) promoter methylation [6]—into the diagnostic and prognostic framework [7,8]. However, significant heterogeneity in clinical outcomes persists within molecularly defined subgroups [9], underscoring the need for additional prognostic biomarkers.

Sidekick Cell Adhesion Molecule 1 (SDK1) is an immunoglobulin superfamily member that mediates homophilic cell-cell adhesion. Originally identified for its role in synaptic connectivity and laminar targeting in the retina [10,11], SDK1 has been implicated in cell adhesion, migration, and tissue morphogenesis. Emerging evidence indicates that cell adhesion molecules play critical roles in tumor biology, including invasion, metastasis, and microenvironment modulation [12,13]. Although SDK1 protein expression in glioma tissue has been documented in the Human Protein Atlas [14], its prognostic significance has not been systematically investigated.

Notably, cell adhesion molecules occupy a central position in a biological axis connecting extracellular matrix (ECM) remodeling, mesenchymal transition, and immune exclusion in glioma. The mesenchymal subtype of glioblastoma—the most aggressive molecular subclass—is characterized by extensive ECM deposition; integrin-mediated signaling; and an immunosuppressive microenvironment enriched in tumor-associated macrophages [15,16,17,18,19,20,21,22,23,24,25,26]. Adhesion molecules can modulate this axis by facilitating tumor cell–ECM interactions, promoting invasive migration through reorganized matrix scaffolds, and shaping immune cell recruitment patterns [27,28,29]. Given SDK1’s established roles in homophilic binding and tissue organization [10], it represents a biologically plausible candidate linking this adhesion–ECM–mesenchymal–immune suppression cascade.

Recent advances in transcriptomic profiling and machine learning-based prognostic models have expanded the landscape of candidate biomarkers in glioma [30,31]. Additionally, spatial transcriptomics and liquid biopsy approaches have begun to reveal intratumoral heterogeneity at unprecedented resolution [32,33]. However, most computationally identified candidates lack multi-cohort validation and integrated functional characterization, limiting their translational utility.

It is important to distinguish between prognostic biomarkers, which provide outcome information independent of treatment; mechanistic drivers, which causally contribute to disease progression; and surrogate transcriptomic markers, which reflect underlying biological programs without necessarily driving them. Based on current evidence, SDK1 is best characterized as a prognostic biomarker that co-occurs with ECM remodeling programs, with its precise mechanistic role requiring further experimental delineation.

In this study, I aimed to (a) evaluate SDK1’s prognostic value in primary glioma using two large independent cohorts; (b) assess whether SDK1 serves as an independent prognostic factor after adjusting for established molecular markers; and (c) characterize the functional and biological context of SDK1 expression through comprehensive bioinformatics analyses, including pathway enrichment, immune microenvironment profiling, and molecular subtype association.

## 2. Results

### 2.1. High SDK1 Expression Is Independently Associated with Poor Overall Survival in Both Cohorts

**Figure 1:**
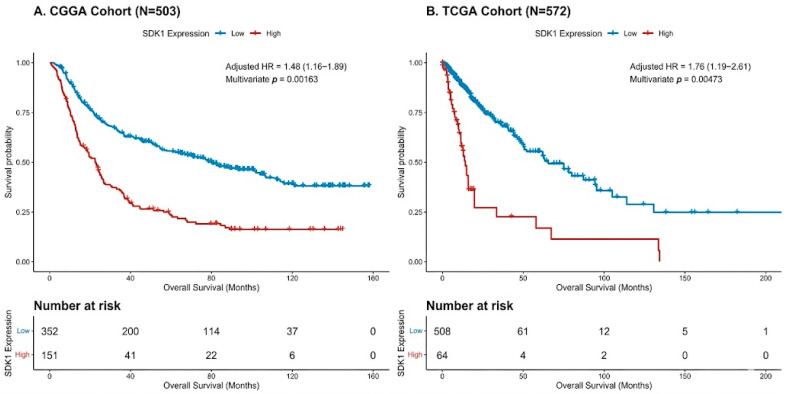
Overall Survival by SDK1 Expression. (**A**) CGGA (N = 503). (**B**) TCGA (N = 572). Number-at-risk tables shown.

**Figure 2:**
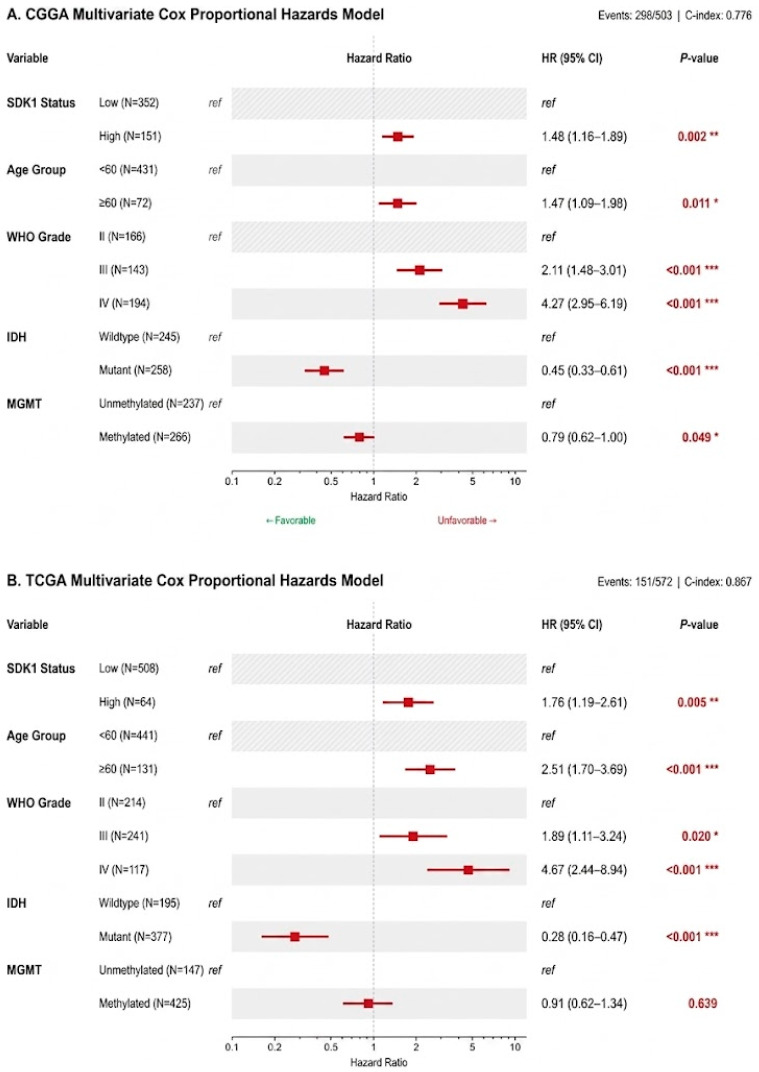
Multivariate Cox Forest Plots. (**A**) CGGA cohort (N = 503). (**B**) TCGA cohort (N = 572). Models include SDK1 expression status (high vs. low [reference]), age group (<60 [reference] vs. ≥60 years), WHO grade (II [reference], III, IV), IDH mutation status (wildtype [reference], mutant), and MGMT promoter methylation (unmethylated [reference], methylated). Hazard ratios with 95% confidence intervals shown. C-indices and AIC values displayed. * *p* < 0.05; ** *p* < 0.01; *** *p* < 0.001.

**Figure 3:**
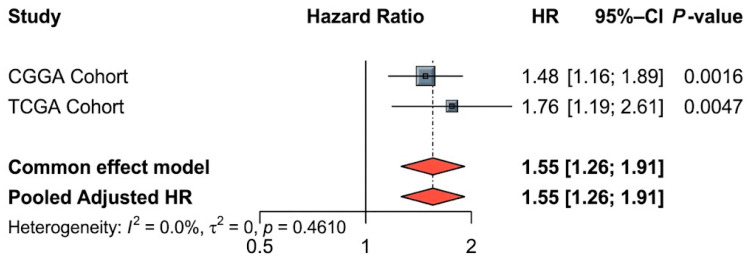
Meta-analysis forest plot pooling SDK1’s adjusted prognostic effect across CGGA and TCGA. Squares denote cohort-specific adjusted HRs from multivariate Cox models (line = 95% CI; size ∝ inverse-variance weight); the diamond shows the fixed-effect pooled estimate (HR = 1.55, 95% CI 1.26–1.91). Between-study heterogeneity was negligible (I^2^ = 0%, *p*-heterogeneity = 0.461). HR > 1 indicates worse survival in SDK1-high tumors.

SDK1-high patients had significantly worse OS in CGGA (log-rank *p* < 0.0001; Figure 1A) and after multivariate adjustment (HR = 1.48, 95% CI 1.16–1.89, *p* = 0.002; Figure 2A). In TCGA, results were consistent (HR = 1.76, 95% CI 1.19–2.61, *p* = 0.005; Figure 1B and Figure 2B). The full model C-indices were 0.776 (CGGA) and 0.879 (TCGA). Meta-analysis yielded pooled HR = 1.55 (95% CI 1.26–1.91, I^2^ = 0%, P-heterogeneity = 0.4610; Figure 3).

The PH assumption was satisfied for SDK1 in both cohorts (CGGA *p* = 0.680; TCGA *p* = 0.603; Supplementary Table S2). In the CGGA cohort, Grade (*p* = 0.007) and IDH (*p* < 0.001) violated the assumption. In the TCGA cohort, IDH (*p* < 0.001) and MGMT (*p* = 0.041) violated the assumption. Because the primary variable of interest (SDK1) satisfied the PH assumption in both cohorts, the standard multivariate Cox model (Figure 2) was retained as the primary analysis. As a pre-specified sensitivity analysis, stratified Cox models—stratifying by the PH-violating variables (Grade and IDH in CGGA; IDH and MGMT in TCGA)—yielded consistent results: HR = 1.40 (95% CI 1.10–1.79, *p* = 0.007) in CGGA and HR = 1.97 (95% CI 1.33–2.94, *p* < 0.001) in TCGA (Supplementary Table S3), confirming robustness.

While the incremental C-index improvement upon adding SDK1 to the base model was modest (ΔCGGA: +0.005; ΔTCGA: +0.005; Supplementary Figure S8A), decision curve analysis demonstrated marginal net benefit of the SDK1-inclusive model at threshold probabilities between 24 and 87% (CGGA) and 33–87% (TCGA), though the absolute improvement was small (Supplementary Figure S9). The continuous NRI was 0.50 (95% CI 0.34–0.68, *p* < 0.001) in CGGA and 0.50 (95% CI 0.29–0.69, *p* < 0.001) in TCGA, indicating improved risk discrimination in a category-free framework. However, the categorical NRI using 20%/50% risk thresholds was not significant (CGGA: −0.02, *p* = 1.00; TCGA: 0.01, *p* = 0.57), consistent with SDK1’s modest incremental C-index improvement (+0.005) and reinforcing its role as a biological stratifier rather than a clinically transformative predictor.

### 2.2. SDK1 Prognostic Association Is Consistent in Grade IV and IDH-Wildtype Subgroups but Attenuated in Lower-Grade and Older Patient Strata

**Figure 4:**
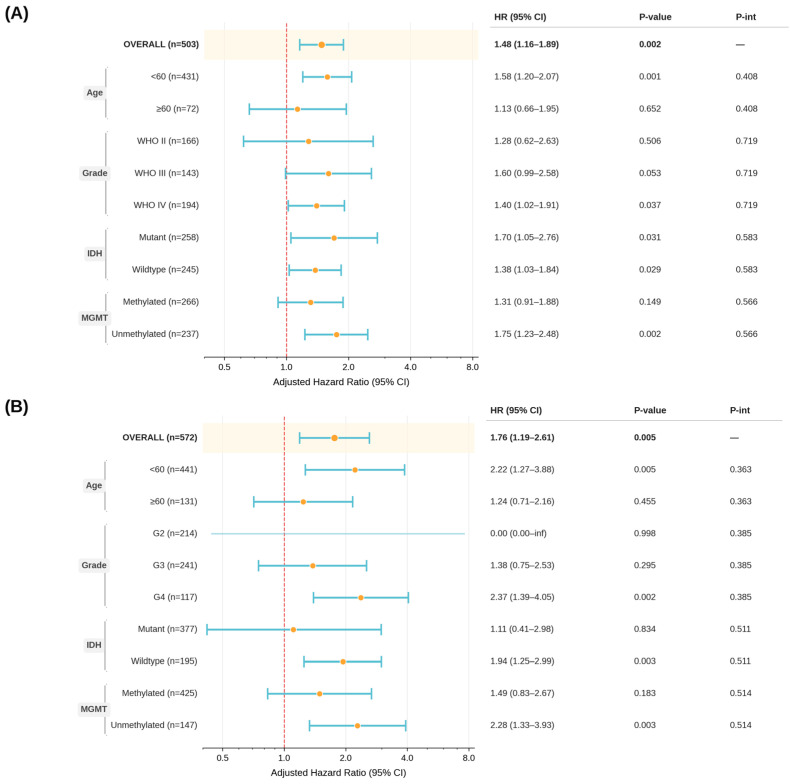
Subgroup forest plot of adjusted hazard ratios for SDK1 for the (**A**) CGGA cohort and (**B**) TCGA cohort, stratified by age, WHO grade, IDH status, and MGMT methylation, with corresponding interaction *p*-values.

Although interaction tests indicated no statistically significant heterogeneity across clinical subgroups (Age P-int = 0.406; Grade P-int = 0.719; IDH P-int = 0.583; MGMT P-int = 0.566), the prognostic strength of SDK1 varied across strata (Figure 4). SDK1 did not reach statistical significance in patients aged ≥ 60 years (HR = 1.13, 95% CI 0.66–1.95, *p* = 0.652), WHO grade II tumors (HR = 1.28, 95% CI 0.62–2.63, *p* = 0.506), WHO grade III tumors (HR = 1.60, 95% CI 0.99–2.58, *p* = 0.053), or MGMT-methylated tumors (HR = 1.31, 95% CI 0.91–1.88, *p* = 0.149). In contrast, SDK1 remained significantly associated with worse overall survival in patients aged <60 years (HR = 1.58, 95% CI 1.20–2.07, *p* = 0.001), WHO grade IV tumors (HR = 1.40, 95% CI 1.02–1.91, *p* = 0.037), IDH-mutant tumors (HR = 1.70, 95% CI 1.05–2.76, *p* = 0.031), IDH-wildtype tumors (HR = 1.38, 95% CI 1.03–1.84, *p* = 0.029), and MGMT-unmethylated tumors (HR = 1.75, 95% CI 1.23–2.48, *p* = 0.002). In the TCGA cohort, SDK1 remained significant in the IDH-wildtype subgroup (HR = 1.94, 95% CI 1.25–2.99, *p* = 0.003) and WHO grade IV (HR = 2.37, 95% CI 1.39–4.05, *p* = 0.002) but did not reach significance in the IDH-mutant subgroup (HR = 1.11, 95% CI 0.41–2.98, *p* = 0.834). These findings suggest that the adverse prognostic association of SDK1 is present across multiple clinical contexts, although its statistical significance is attenuated in some subgroups, likely due to a smaller sample size or subgroup-specific biological heterogeneity.

### 2.3. Cross-Validated DEGs Are Enriched in Extracellular Matrix Organization and Focal Adhesion Pathways

**Figure 5:**
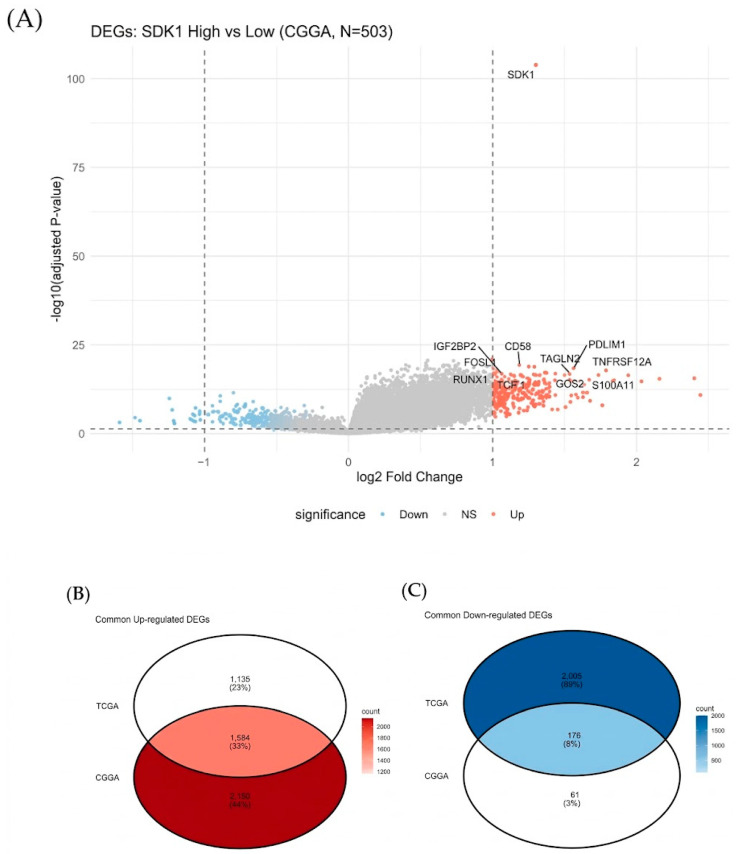
Cross-Validated DEGs. (**A**) Volcano plot. (**B**,**C**) Venn diagrams showing overlap between CGGA and TCGA DEGs. Cross-validation yielded 1584 up-regulated and 176 down-regulated common DEGs. The predominance of upregulated genes likely reflects the transcriptionally active and mesenchymal-like phenotype of SDK1-high tumors.

**Figure 6:**
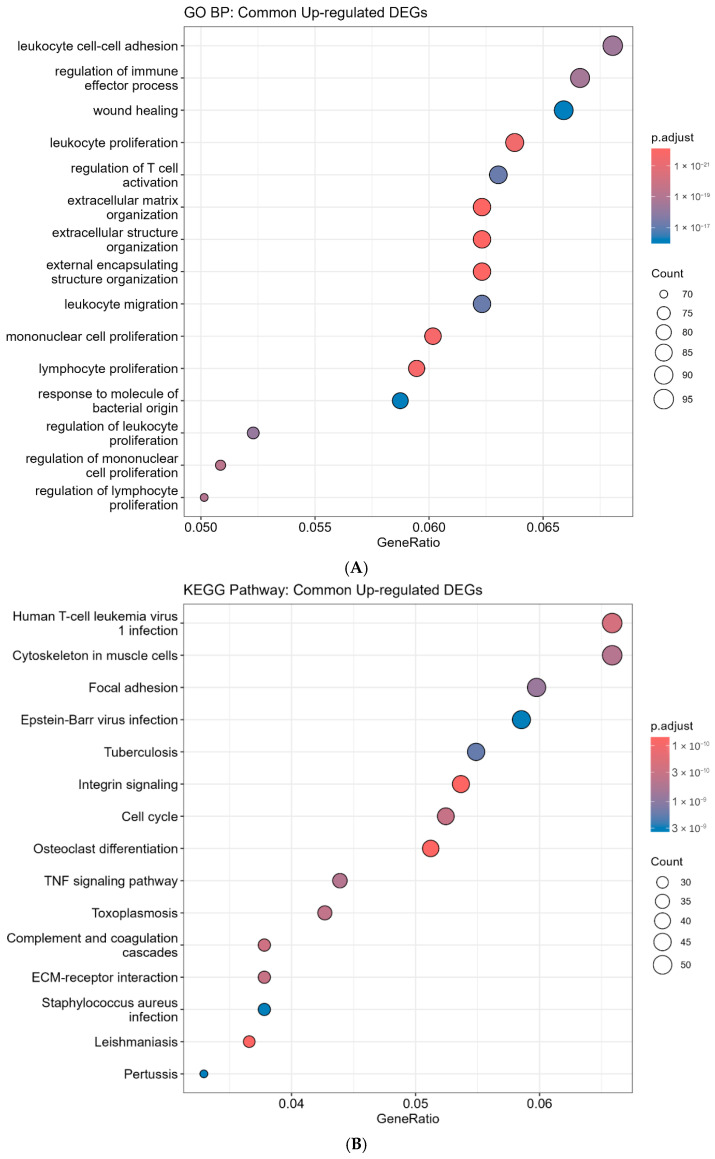
Pathway Enrichment Analysis. (**A**) GO Biological Process enrichment. (**B**) KEGG pathway enrichment. Key pathways: ECM organization, focal adhesion, integrin signaling, and ECM-receptor interaction.

Cross-validation yielded 1584 up-regulated and 176 down-regulated common DEGs (Figure 5), reflecting the predominance of genes positively correlated with SDK1 expression. GO analysis revealed enrichment in ECM organization (P-adj < 10^−16^), wound healing, chemotaxis, and cell-substrate adhesion (Figure 6A). KEGG identified focal adhesion, integrin signaling, ECM-receptor interaction, PI3K-Akt signaling [33], and proteoglycans in cancer (Figure 6B)—consistent with SDK1’s adhesion molecule function [10,12,34].

To directly link SDK1 to established mesenchymal structural frameworks, I performed correlation analyses between SDK1 expression and (a) a COL1A1-associated mesenchymal program score and (b) an oncostream-related gene signature score [34,35]. The mesenchymal program score was computed as the mean z-scored expression of COL1A1, COL1A2, COL3A1, COL5A1, FN1, VIM, ACTA2, TAGLN, POSTN, and SPARC [15,34,36]. SDK1 expression showed weak but statistically significant positive correlations with the mesenchymal program score in both CGGA (Spearman R = 0.12, *p* = 0.008) and TCGA (R = 0.15, *p* < 0.001; Supplementary Figure S10). The oncostream signature score was also weakly correlated with SDK1 in CGGA (R = 0.16, *p* < 0.001), though the correlation was marginal in TCGA (R = 0.086, *p* = 0.039; Supplementary Figure S11). While statistically significant, the modest effect sizes (R = 0.08–0.16) indicate that SDK1 shares only a small proportion of variance with these mesenchymal and invasion programs, suggesting that SDK1 may be a peripheral co-regulated marker within this transcriptional landscape rather than a core component.

### 2.4. SDK1-High Tumors Exhibit an Immunosuppressive Transcriptomic Profile with Elevated Immune Checkpoint Expression

The following immune associations are correlative and derived from ssGSEA-based bulk RNA deconvolution, which estimates transcriptomic programs rather than cell counts and cannot fully resolve tumor-intrinsic versus stromal contributions [37]. These caveats should be considered when interpreting the results below.

**Figure 7:**
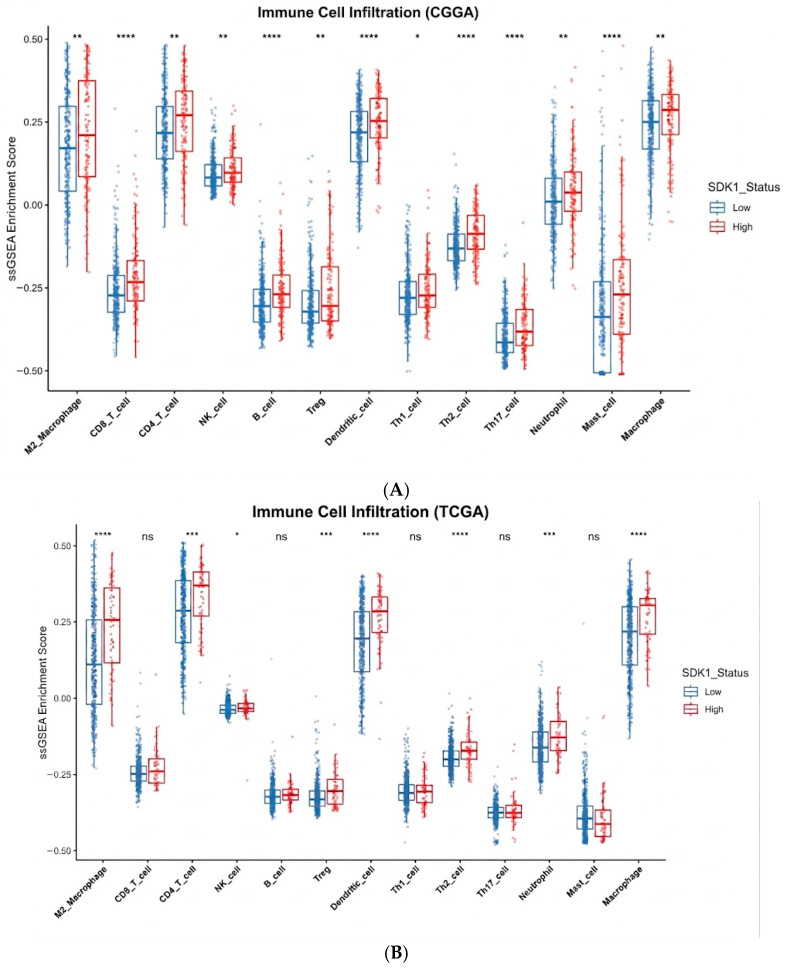
Immune Cell Infiltration by ssGSEA. (**A**) CGGA cohort. (**B**) TCGA cohort. Comparison of 13 immune cell type signatures between SDK1-high and SDK1-low groups. * *p* < 0.05; ** *p* < 0.01; *** *p* < 0.001, **** *p* < 0.0001, ns = non-significant.

**Figure 8:**
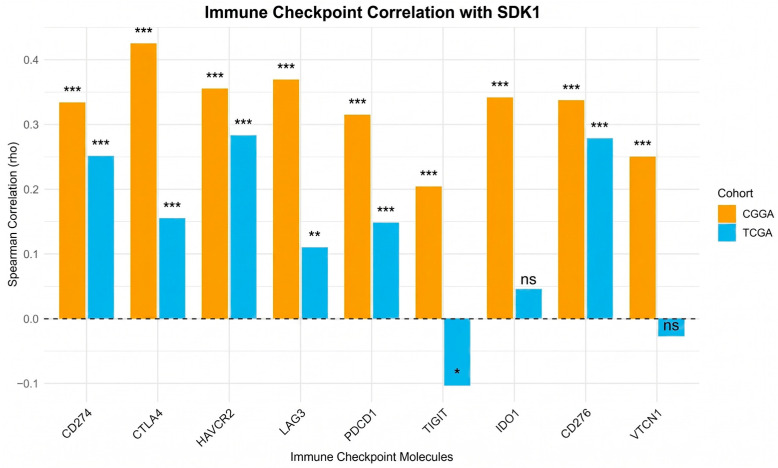
Immune Checkpoint Correlation. Spearman correlation coefficients between SDK1 expression and immune checkpoint molecules (CD274/PD-L1, CTLA4, HAVCR2/TIM-3, LAG3, PDCD1/PD-1, TIGIT, IDO1, CD276, VTCN1) in CGGA and TCGA cohorts. * *p* < 0.05; ** *p* < 0.01; *** *p* < 0.001, ns = non-significant.

SDK1-high tumors exhibited elevated M2 macrophage signatures in both cohorts (CGGA *p* < 0.01; TCGA *p* < 0.0001), with reduced CD8+ T cell signatures in CGGA (*p* < 0.0001) but not in TCGA (*p* = ns), reduced CD4+ T cell signatures in both cohorts (CGGA *p* < 0.01; TCGA *p* < 0.001), and reduced NK cell signatures in CGGA (*p* < 0.01) but not in TCGA (*p* = ns; Figure 7). B cell signatures were significantly reduced in CGGA (*p* < 0.0001) but did not reach significance in TCGA (*p* = ns). Treg signatures were elevated in SDK1-high tumors in both cohorts (CGGA *p* < 0.01; TCGA *p* < 0.001). Dendritic cell signatures were also elevated in SDK1-high tumors (both *p* < 0.0001). Th1 signatures showed a modest difference in CGGA (*p* < 0.05) but not in TCGA (*p* = ns). SDK1 expression showed significant positive correlations with multiple immune checkpoint molecules in both cohorts, including CD274/PD-L1 (CGGA rho = 0.34, *p* < 0.001; TCGA rho = 0.25, *p* < 0.001), CTLA4 (CGGA rho = 0.43, *p* < 0.001; TCGA rho = 0.15, *p* < 0.001), HAVCR2/TIM-3 (CGGA rho = 0.36, *p* < 0.001; TCGA rho = 0.29, *p* < 0.001), LAG3 (CGGA rho = 0.37, *p* < 0.001; TCGA rho = 0.11, *p* < 0.01), and PDCD1/PD-1 (CGGA rho = 0.33, *p* < 0.001; TCGA rho = 0.15, *p* < 0.001) (Figure 8). TIGIT showed a positive correlation in CGGA (rho≈0.21, *p* < 0.001) but a weak negative correlation in TCGA (rho ≈ −0.09, *p* < 0.05). IDO1 was positively correlated in CGGA (*p* < 0.001) but did not reach significance in TCGA, and VTCN1 was positively correlated in CGGA (*p* < 0.001) but not significant in TCGA. These represent co-occurring transcriptomic signatures [35,36,38]; bulk RNA deconvolution cannot fully resolve tumor-intrinsic versus stromal contributions [39], and single-cell or spatial transcriptomic validation is needed.

To assess whether the immunosuppressive profile of SDK1-high tumors is independent of mesenchymal transition, I performed partial correlation analyses controlling for the COL1A1-associated mesenchymal program score. After adjustment for the mesenchymal program score, SDK1 maintained significant partial correlations with M2 macrophage signatures in both cohorts (CGGA: partial rho = 0.093, *p* = 0.038; TCGA: partial rho = 0.188, *p* < 0.001), indicating that this association is partially independent of the mesenchymal program. CD8+ T cell, NK cell, B cell, and dendritic cell correlations were also maintained after adjustment in CGGA (all *p* < 0.05). Notably, in TCGA, several immune cell associations that were non-significant in the unadjusted analysis (Treg, CD8+ T cell, NK cell, and B cell) became statistically significant after mesenchymal adjustment (partial rho ranging from −0.084 to −0.158, all *p* < 0.05), suggesting a statistical suppressor effect in which the mesenchymal program had been masking these immune associations. The Treg association showed cohort-dependent behavior: non-significant in both unadjusted and adjusted analyses in CGGA but revealing a significant negative partial correlation in TCGA (partial rho = −0.158, *p* < 0.001) (Supplementary Table S6).

### 2.5. SDK1 Expression Shows a Nominally Higher Trend in the Mesenchymal Subtype Without Overall Significance

SDK1’s mesenchymal subtype association provides additional context. However, the overall Kruskal–Wallis test was not statistically significant (*p* = 0.217), and the observed nominal difference in the mesenchymal subgroup (*p* = 0.04) should be interpreted cautiously and considered exploratory. Mesenchymal GBMs feature NF1 loss, NF-κB activation, and ECM gene expression [30,37,40].

**Figure 9:**
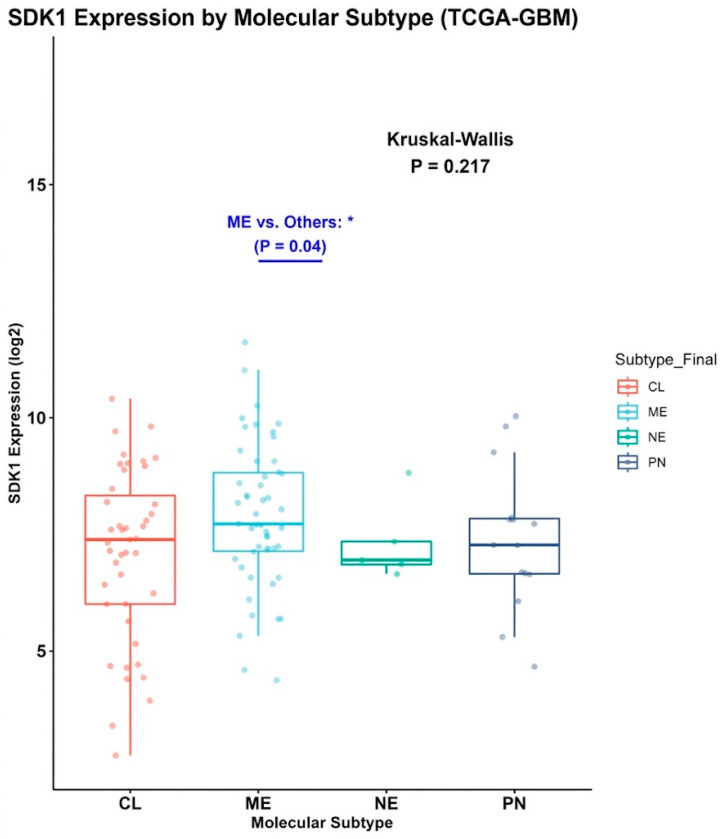
SDK1 Expression by Molecular Subtype in TCGA-GBM. Kruskal–Wallis *p* = 0.217. Mesenchymal vs. Others: *p* = 0.04. (CL: classical, ME: mesenchymal, NE: neural, PN: proneural). * Nominally elevated SDK1 expression in the mesenchymal subtype compared to other subtypes (exploratory pairwise comparison).

The following subtype analysis is exploratory and restricted to the GBM subset of the TCGA cohort; it should not be generalized to the full primary glioma population. SDK1 expression did not differ significantly across TCGA-GBM subtypes by overall Kruskal–Wallis test (*p* = 0.217; Figure 9), although an exploratory pairwise comparison suggested a nominal difference between the mesenchymal subtype and other subtypes (*p* = 0.04), which did not indicate a significant overall subtype effect. The mesenchymal subtype is characterized by aggressive biology, ECM gene expression, and poor prognosis [30,40,41]. Future co-expression analysis with mesenchymal drivers (NF1, NF-κB, STAT3) [37,40] would clarify SDK1’s position within this program.

### 2.6. SDK1 Is Peripherally Positioned in an ECM- and Cell Cycle-Centered Interaction Network

**Figure 10:**
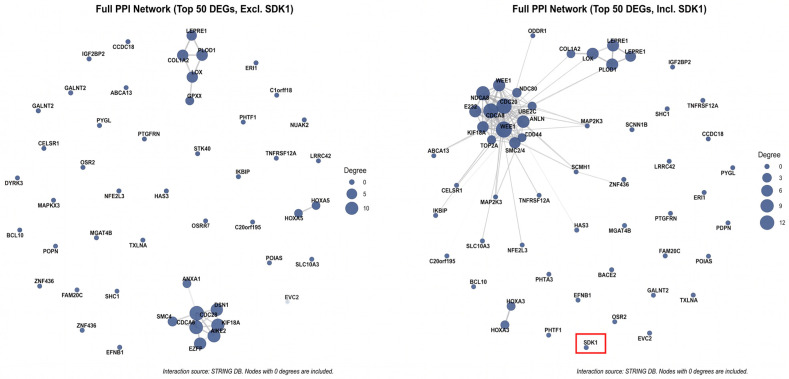
Protein-Protein Interaction (PPI) Network. STRING network of the top 50 cross-validated DEGs (excluding SDK1). Network including SDK1; SDK1 positioned peripherally. Hub genes: *COL6A2*, *LOX*, *LEPRE1*, *PLOD1*, *CDC20*, *CDCA8*, *KIF2C*, *WEE1*. Several hub genes identified in the PPI network, including *COL6A2*, *LOX*, and *PLOD1*, are involved in collagen organization and extracellular matrix maturation, supporting the notion that SDK1-high tumors are associated with matrix remodeling processes characteristic of mesenchymal-like glioma biology.

PPI analysis of the top 50 cross-validated DEGs (excluding SDK1) revealed a network (Figure 10). Hub genes (by node degree) included *COL6A2*, *LOX*, *LEPRE1*, *PLOD1*, *CDC20*, *CDCA8*, *KIF2C*, and *WEE1*. When SDK1 was included in the network (replacing the lowest-ranked DEG), SDK1 was positioned peripherally with limited direct connections (Figure 10), consistent with its role as a co-expressed marker rather than a central physical interaction hub within the ECM/adhesion network. The network architecture centered on ECM remodeling, cell cycle regulation, and adhesion signaling, reinforcing biological coherence.

The peripheral positioning of SDK1 in the PPI network, combined with its strong transcriptomic co-expression with ECM and mesenchymal genes but lack of direct physical interaction evidence, suggests that SDK1 is most likely a co-regulated marker within the broader mesenchymal/ECM remodeling program rather than a central driver or downstream consequence. SDK1 may be transcriptionally co-regulated with ECM components through shared upstream regulators (e.g., NF-κB and STAT3) that drive the mesenchymal program [16,17], while its adhesion molecule function could contribute to ancillary roles in cell–ECM interaction and migration. Distinguishing between these possibilities will require experimental approaches such as SDK1 knockdown/overexpression with assessment of ECM gene expression changes, invasion assays, and in vivo tumor models.

### 2.7. SDK1-High Tumors Do Not Differ in Tumor Mutational Burden

To assess whether SDK1’s prognostic association is related to genomic instability, I compared tumor mutational burden (TMB) between SDK1-high and SDK1-low groups in the TCGA cohort. TMB was defined as the total number of non-synonymous somatic mutations per sample. SDK1-high tumors showed a significantly higher TMB compared to SDK1-low tumors, although the absolute difference in median values was modest (Wilcoxon *p* = 4.3 × 10^−5^; Supplementary Figure S12). However, this association was not consistently observed across all subgroups, and there was no significant correlation between continuous SDK1 expression and TMB (Spearman rho = 0.038, *p* = 0.368). Most importantly, the SDK1 × TMB interaction term in a multivariate Cox model was not significant (*p* = 0.129; Supplementary Table S7), indicating that TMB does not modify SDK1’s prognostic effect. These results suggest that while SDK1 expression may be associated with TMB, its role as a prognostic biomarker remains independent of the tumor’s overall mutational load.

## 3. Discussion

In this multi-cohort study, I systematically evaluated SDK1 as a prognostic biomarker in primary glioma, demonstrating that high expression is associated with worse OS in both CGGA (HR = 1.48) and TCGA (HR = 1.76), with a pooled HR = 1.55 and zero inter-cohort heterogeneity. While SDK1 expression in glioma has been documented [14], to our knowledge, this study represents the first multi-cohort validation of its independent prognostic value combined with integrated functional characterization. I position our contribution as the systematic demonstration that SDK1 independently stratifies glioma prognosis within a biologically plausible adhesion–ECM–immune suppression context.

Several prognostic biomarkers have been proposed in glioma beyond the established IDH/MGMT framework, including YKL-40 [21], ATRX loss, TERT promoter mutations [22], and various gene expression signatures [30,31]. However, many lack systematic multi-cohort validation or integrated biological characterization. SDK1 differs in that it occupies a defined position at the intersection of cell adhesion, ECM remodeling, and immune modulation—providing not only prognostic information but also a biologically interpretable link to tumor microenvironment organization. As established in the Introduction, SDK1 is best characterized as a prognostic biomarker and co-regulated transcriptomic marker within the mesenchymal program, rather than a demonstrated mechanistic driver.

Several lines of evidence support SDK1 as a biologically meaningful marker rather than a purely incidental association. First, it maintained significance after IDH/Grade/Age/MGMT adjustment and in stratified Cox models. Second, in the CGGA cohort, it remained significantly associated with outcome within the IDH-mutant subgroup (HR = 1.70, 95% CI 1.05–2.76, *p* = 0.031), although this was not replicated in TCGA (HR = 1.11, *p* = 0.834), suggesting potential cohort-dependent variation. Third, the functional profile—ECM remodeling; integrin signaling; and focal adhesion—is consistent with its adhesion molecule identity [10]. Nonetheless, the moderate effect size (HR 1.4–1.8) and modest incremental improvement in C-index upon adding SDK1 to the base model (ΔCGGA: +0.005; ΔTCGA: +0.005; Supplementary Figure S8A) indicate that SDK1 is best viewed as a biological stratifier rather than a standalone clinical predictor. Although the correlations between SDK1 and COL1A1-associated mesenchymal program scores were statistically significant in both cohorts, the modest effect sizes (R = 0.12–0.15) indicate that SDK1 shares limited variance with the core mesenchymal transcriptional program. Similarly, the weak correlation with oncostream signatures (R = 0.086–0.16) provides only preliminary evidence for SDK1’s involvement in spatially organized invasion. These findings are consistent with SDK1’s peripheral positioning in the PPI network and support its characterization as a co-regulated marker at the boundary of the mesenchymal program, rather than a central component. Spatial transcriptomic validation will be essential to determine whether SDK1 has any direct role in oncostream formation. The full model nonetheless demonstrated acceptable to strong discriminative performance across both cohorts, with time-dependent AUCs of 0.767–0.856 in CGGA and 0.867–0.946 in TCGA at 2-, 3-, and 5-year timepoints (Supplementary Figure S8B,C).

Cell adhesion molecules frequently function at the interface between tumor cells and the extracellular matrix, shaping both invasive behavior and immune microenvironment organization. The enrichment of extracellular matrix–related pathways together with increased M2 macrophage and Treg signatures observed in SDK1-high tumors suggests that SDK1 may mark a transcriptional program associated with mesenchymal-like tumor–microenvironment interactions.

The partial correlation analyses revealed that most immune associations of SDK1 are not primarily mediated through the mesenchymal program. In CGGA, M2 macrophage, CD8+ T cell, NK cell, B cell, and dendritic cell associations all persisted after mesenchymal adjustment, with only modest attenuation. In TCGA, a suppressor effect was observed: several originally non-significant immune associations became significant after mesenchymal adjustment, suggesting that the mesenchymal program had been acting as a confounder masking these relationships. It should be noted that because the SDK1–mesenchymal correlation was itself weak (R = 0.12–0.15, explaining only 1–2% of variance), the mesenchymal adjustment has limited statistical leverage. The observed suppressor effects in TCGA may reflect the removal of a small confounding pathway rather than a strong mediating relationship.

The immunosuppressive transcriptomic profile of SDK1-high tumors is of translational interest. Elevated M2 macrophage and Treg signatures with reduced CD4+ T cell programs were observed in both cohorts, while CD8+ T cell and NK cell reductions were significant only in CGGA but not in TCGA, coupled with upregulated checkpoints (PD-L1, CTLA-4, TIM-3, LAG-3), suggesting an immune-evasive landscape [35,36,38]. Notably, B cell signatures were significantly reduced only in CGGA but not in TCGA, suggesting that this association may be cohort-dependent or influenced by platform differences. These associations are correlative; whether SDK1 causally drives immunosuppression, results from it, or co-occurs within the same aggressive program cannot be determined from these data. These findings raise the hypothesis that SDK1 expression may be linked to immune evasion programs [42,43], warranting further investigation in studies of immunotherapy response. The single-cell RNA-seq analysis (Supplementary Figure S7) provides preliminary evidence that SDK1 is expressed in monocyte/macrophage populations and subsets of malignant cells, consistent with the bulk RNA associations. Spatial transcriptomic approaches such as Visium, MERFISH, or multiplexed IHC (e.g., CODEX) would be particularly informative for determining (a) whether SDK1 protein localizes to the tumor–stroma interface consistent with its adhesion function, (b) whether SDK1+ tumor cells spatially co-localize with M2 macrophages and Tregs, and (c) whether SDK1 expression overlaps with oncostream structures. We recommend these as priority experiments for subsequent validation studies.

SDK1’s mesenchymal subtype association provides additional context. However, the overall Kruskal–Wallis test was not statistically significant (*p* = 0.217), and the observed nominal difference in the mesenchymal subgroup (*p* = 0.04) should be interpreted cautiously and considered exploratory. Mesenchymal GBMs feature NF1 loss, NF-κB activation, and ECM gene expression [30,37,40]. PPI hub genes *COL6A2*, *LOX*, and *PLOD1* are established ECM components. SDK1 remained significantly associated with outcome in the IDH-wildtype subgroup in both cohorts and in the IDH-mutant subgroup in CGGA but not in TCGA, suggesting that its prognostic association may vary by molecular context. In GBM, it may reflect the mesenchymal commitment degree, while in lower-grade tumors, it may identify subsets with enhanced invasion potential portending progression. Non-significant interaction tests argue against a subtype-specific epiphenomenon.

This study has several limitations. First, all analyses are computational, establishing association, not causation. Experimental validation through SDK1 knockdown/overexpression with functional assays and in vivo models is essential; I frame findings as hypothesis-generating. Second, SDK1 expression has been catalogued in HPA [14]; our novelty lies in systematic multi-cohort prognostic validation and integrated functional characterization. Third, complete-case analysis may introduce selection bias if excluded patients differ systematically. Fourth, the reliance on maximally selected rank statistics (maxstat) resulted in disparate cutoff proportions between the CGGA (30.0% high) and TCGA (11.2% high) cohorts. The maxstat approach was selected because it provides an objective, data-driven threshold without requiring arbitrary percentile selection; however, this carries a risk of overfitting to cohort-specific distributions. The pre-specified sensitivity analysis using a fixed 30th percentile cutoff in TCGA confirmed robustness (HR = 2.33, *p* = 2.14 × 10^−7^), and the meta-analysis showed no detectable heterogeneity (I^2^ = 0%). For clinical translation, standardized IHC scoring algorithms or pre-defined expression percentile cutoffs validated in independent cohorts are recommended. Fifth, our subgroup analyses revealed that SDK1’s prognostic impact is not uniform across all histologies; it failed to reach statistical significance in WHO grade II gliomas (HR = 1.28, 95% CI 0.62–2.63, *p* = 0.506) and in several other subgroups. Therefore, SDK1 is best positioned as a biological stratifier in high-grade (Grade III/IV) glioma contexts, rather than a universal clinical decision tool for all gliomas. Sixth, although the 2021 WHO classification incorporates 1p/19q codeletion status as a defining molecular marker for oligodendroglioma, this variable was not included in our multivariable models due to incomplete availability across both cohorts. This is an important limitation that may have resulted in residual confounding, particularly in lower-grade glioma subgroups where 1p/19q codeletion defines oligodendroglioma versus astrocytoma. Future studies should incorporate 1p/19q status as a covariate. Seventh, CGGA and TCGA datasets were analyzed independently rather than merged, avoiding direct cross-platform harmonization challenges. Each cohort used its own internal normalization (CGGA: RSEM; TCGA: Firehose RSEM log2). The consistent direction and magnitude of effects, confirmed by meta-analysis with I^2^ = 0%, support robustness despite platform differences, though formal batch correction in a harmonized framework would further strengthen results. Eighth, ssGSEA-based immune profiling from bulk RNA cannot resolve tumor vs. stromal contributions and estimates transcriptomic programs rather than cell counts [37]; single-cell or multiplex IHC validation is needed. Ninth, SDK1’s druggability is unexplored; as a transmembrane Ig superfamily member, antibody-based targeting is theoretically feasible, but therapeutic tractability and neural tissue toxicity remain speculative.

## 4. Materials and Methods

### 4.1. Study Population and Data Sources

Gene expression data and clinical information were obtained from the Chinese Glioma Genome Atlas (CGGA, http://www.cgga.org.cn) [36,44] and The Cancer Genome Atlas (TCGA) via the UCSC Xena platform [38,45,46]. The CGGA dataset comprised two RNA-seq sub-cohorts (CGGA-325 and CGGA-693). The TCGA dataset included combined LGG and GBM samples (Firehose RSEM log2-normalized) [9,39,47]. For TCGA, only primary tumor samples (sample code “01”) were retained. Clinical variable names and categorical labels were standardized using regular expression-based text matching to resolve inconsistencies. All recurrent and secondary tumors were excluded. A complete-case analysis framework was adopted: patients with missing data in any core variable (overall survival, vital status, age, WHO grade, IDH status, or MGMT methylation) were excluded. Age was also categorized (<60 vs. ≥60 years). Final cohorts comprised 503 (CGGA) and 572 (TCGA) patients.

SDK1 expression did not differ between CGGA sub-cohorts (Wilcoxon *p* = 0.125; Supplementary Figure S1). Grade distribution differed (*p* = 0.003), but SDK1 status (*p* = 0.289), IDH (*p* = 0.505), and age (*p* = 0.883) were comparable (Supplementary Table S1). The SDK1 HR was consistent across sub-cohorts (CGGA-693: adjusted HR = 1.42, 95% CI 1.02–1.98; CGGA-325: adjusted HR = 1.48, 95% CI 1.01–2.18; pooled HR = 1.45, 95% CI 1.13–1.86, I^2^ = 0%; Supplementary Figure S2). Subsequently, in the cross-cohort meta-analysis between CGGA and TCGA, no significant heterogeneity was detected (I^2^ = 0%, P-heterogeneity = 0.461; Figure 3). The final CGGA cohort comprised 503 patients (352 low, 151 high); TCGA comprised 572 (508 low, 64 high). SDK1-high patients were significantly enriched for Grade IV (CGGA: 58.3% vs. 30.1%; TCGA: 43.8% vs. 17.5%) and IDH-wildtype status (CGGA: 73.5% vs. 38.1%; TCGA: 75.0% vs. 28.9%) (all *p* < 0.001; Table 1), necessitating multivariate adjustment.

### 4.2. Optimal Cutoff Determination

Optimal SDK1 expression cutoffs were determined independently in each cohort using maximally selected rank statistics (maxstat) [48] via the surv_cutpoint function in survminer [49]. This algorithm iteratively evaluates all possible thresholds, selecting the cutpoint maximizing the standardized log-rank statistic. The maxstat approach was selected because it provides an objective threshold without requiring arbitrary percentile selection; however, the resulting divergent high-risk proportions between cohorts (CGGA 30.0%, TCGA 11.2%) represent a known limitation and underscore the need for standardized cutoff development for clinical application. A pre-specified sensitivity analysis using the upper 30th percentile was performed in TCGA. Maxstat identified cutpoints of 2.21 (CGGA) and 8.79 (TCGA; Supplementary Figure S3). Sensitivity analysis in TCGA using the 30th percentile cutoff confirmed robustness (HR = 2.33, *p* = 2.14 × 10^−7^).

### 4.3. Survival Analysis

Overall survival (OS) was defined as time from diagnosis to death or last follow-up. Kaplan–Meier curves were compared using log-rank tests [42]. Multivariate Cox proportional hazards models [43] included SDK1 status, age, WHO grade, IDH status, and MGMT methylation. The proportional hazards assumption was assessed using Schoenfeld residuals [50]. Variables violating the assumption were handled via stratification. The standard multivariate Cox model was retained as the primary analysis, provided that SDK1 satisfied the PH assumption; stratified Cox models were performed as pre-specified sensitivity analyses. Results were pooled using fixed-effect meta-analysis [51].

Decision curve analysis (DCA) was performed using the dcurves R package to assess the clinical utility of the SDK1-inclusive model compared to the base model across a range of threshold probabilities. Categorical net reclassification improvement (NRI) was calculated using the nricens package, with event/non-event categories defined at 3-year follow-up.

### 4.4. Subgroup Analysis

Subgroup analyses were stratified by age, WHO grade, IDH status, and MGMT methylation. Interaction terms (SDK1_Status × Clinical_Variable) were tested. Subgroups with <3 events in the SDK1-high group were excluded from forest plots with footnote documentation.

### 4.5. Differential Gene Expression Analysis

DEGs between SDK1-high and SDK1-low groups were identified independently using limma [52]. Significance required |log2FC| > 1 and adjusted *p* < 0.05. Cross-validated DEGs were defined as genes significant in both cohorts with consistent direction.

### 4.6. Pathway Enrichment Analysis

GO Biological Process and KEGG analyses were performed on cross-validated DEGs using clusterProfiler [53] with org.Hs.eg.db annotation.

### 4.7. Mesenchymal Program and Oncostream Signature Analysis

A COL1A1-associated mesenchymal program score was calculated as the mean of z-scored expression values for COL1A1, COL1A2, COL3A1, COL5A1, FN1, VIM, ACTA2, TAGLN, POSTN, and SPARC [15,34,36]. An oncostream-related signature score was computed using SERPINE1, THBS1, TAGLN, LGALS1, ANXA2, S100A11, and MYL9 [35]. Spearman correlations between SDK1 expression and these composite scores were assessed. Partial correlation analyses controlling for the mesenchymal program score were performed using the ppcor R package to assess the independence of immune associations from mesenchymal transition.

### 4.8. Immune Microenvironment Analysis

Immune cell infiltration was estimated using ssGSEA [54] via GSVA [55] with predefined gene signatures for 13 immune cell types based on established marker sets [56,57]. Correlations between SDK1 and immune checkpoint genes (CD274, PDCD1, CTLA4, HAVCR2, LAG3, TIGIT, IDO1, CD276, VTCN1) were assessed using Spearman coefficients.

### 4.9. Molecular Subtype Analysis

TCGA-GBM molecular subtypes (Classical, Mesenchymal, Neural, and Proneural) were obtained via TCGAbiolinks [58], based on Verhaak et al. [15] and Ceccarelli et al. [9]. SDK1 was compared across subtypes using Kruskal–Wallis with pairwise Wilcoxon tests. This analysis is exploratory and restricted to the GBM subset.

### 4.10. Tumor Mutational Burden Analysis

Somatic mutation data for the TCGA cohort were obtained from the NCI Genomic Data Commons (GDC). Tumor mutational burden (TMB) was calculated as the total number of non-synonymous somatic mutations per sample. Comparisons between SDK1-high and SDK1-low groups were performed using Wilcoxon rank-sum tests. Correlation between continuous SDK1 expression and TMB was assessed using Spearman coefficients. An interaction term (SDK1 × TMB) was tested in a multivariate Cox model.

### 4.11. Protein-Protein Interaction Network

PPI networks were constructed using STRING (version 11.5, score >0.4) [59]. Top 50 cross-validated DEGs were used. Hub genes were ranked by node degree.

### 4.12. Statistical Software

All statistical analyses and visualizations were performed using R software (version 4.5.2; R Foundation for Statistical Computing, Vienna, Austria), with figures generated using ggplot2 [60]. Survival analyses were conducted using the survival and survminer packages. The optimal cutoff values for continuous gene expression were determined using maximally selected rank statistics via the maxstat package. The predictive accuracy of the prognostic models was evaluated using the concordance index (C-index) and time-dependeSSnt receiver operating characteristic (ROC) curve analysis via the timeROC package. Meta-analysis of hazard ratios was performed using the meta package. All statistical tests were two-sided, and a *p*-value < 0.05 was considered statistically significant. The analytical codes used in this study are available from me upon reasonable request.

## 5. Conclusions

In conclusion, this multi-cohort integrative analysis demonstrates that elevated SDK1 expression is consistently associated with poorer survival outcomes in glioma across independent datasets, while providing modest but reproducible incremental prognostic value beyond established clinical and molecular markers. Functional and network analyses suggest that SDK1 is not a central oncogenic driver but rather a peripheral, co-regulated transcriptomic marker linked to extracellular matrix remodeling, mesenchymal transition programs, and immunosuppressive tumor microenvironment features. Importantly, its associations with mesenchymal signatures, oncostream-related gene programs, and immune infiltration patterns were statistically significant but relatively weak, supporting a model in which SDK1 reflects broader tumor ecosystem states rather than acting as a primary regulator. Although the clinical utility of SDK1 as a standalone biomarker remains limited, its consistent cross-cohort performance and biological associations indicate potential value as an adjunctive marker for refined risk stratification and for characterizing tumor microenvironmental heterogeneity. Future studies incorporating spatial transcriptomics and single-cell validation will be essential to clarify its mechanistic role and translational applicability.

## Figures and Tables

**Table 1:** Clinical and molecular characteristics of CGGA (N = 503) and TCGA (N = 572) cohorts stratified by SDK1 expression status. All *p* < 0.001 for Grade IV and IDH-wildtype enrichment. *p*-value was calculated via Welch’s two-sample t-test and Pearson’s Chi-squared test.

Clinical Characteristics	CGGA: SDK1 Low	CGGA: SDK1 High	CGGA *p*-Value	TCGA: SDK1 Low	TCGA: SDK1 High	TCGA *p*-Value
N	352	151		508	64	
Age (mean ± SD)	43.78 (12.80)	46.55 (12.54)	0.026	45.83 (14.88)	54.23 (16.61)	<0.001
Age_Group (%)			0.600			0.001
<60	304 (86.4)	127 (84.1)		403 (79.3)	38 (59.4)	
≥60	48 (13.6)	24 (15.9)		105 (20.7)	26 (40.6)	
Grade (%)			<0.001			<0.001
Grade II	142 (40.3)	24 (15.9)		212 (41.7)	2 (3.1)	
Grade III	104 (29.5)	39 (25.8)		207 (40.7)	34 (53.1)	
Grade IV	106 (30.1)	88 (58.3)		89 (17.5)	28 (43.8)	
IDH (%)			<0.001			<0.001
Mutant	218 (61.9)	40 (26.5)		361 (71.1)	16 (25.0)	
Wildtype	134 (38.1)	111 (73.5)		147 (28.9)	48 (75.0)	
MGMT (%)			0.009			<0.001
Methylated	200 (56.8)	66 (43.7)		393 (77.4)	32 (50.0)	
Unmethylated	152 (43.2)	85 (56.3)		115 (22.6)	32 (50.0)	

## Data Availability

The publicly available datasets analyzed in this study are available from the Chinese Glioma Genome Atlas (CGGA; http://www.cgga.org.cn) and The Cancer Genome Atlas (TCGA) via the UCSC Xena platform (https://xenabrowser.net/, accessed on 7 March 2026) and the NCI Genomic Data Commons (https://portal.gdc.cancer.gov/). The analytical codes used in this study are available from the corresponding author upon reasonable request.

## Supplementary Materials

The following supporting information can be downloaded at: https://www.mdpi.com/article/10.3390/ijms27104199/s1.

## Abbreviations

The following abbreviations are used in this manuscript:

SDK1	Sidekick Cell Adhesion Molecule 1
CGGA	Chinese Glioma Genome Atlas
TCGA	The Cancer Genome Atlas
OS	Overall Survival
HR	Hazard Ratio
CI	Confidence Interval
IDH	Isocitrate Dehydrogenase
MGMT	O6-Methylguanine-DNA Methyltransferase
GBM	Glioblastoma Multiforme
LGG	Low-Grade Glioma
ECM	Extracellular Matrix
DEG	Differentially Expressed Gene
GO	Gene Ontology
KEGG	Kyoto Encyclopedia of Genes and Genomes
ssGEA	Single-Sample Gene Set Enrichment Analysis
PPI	Protein-Protein Interaction
TME	Tumor Microenvironment
PD-L1	Programmed Death-Ligand 1
PD-1	Programmed Cell Death Protein 1
CTLA-4	Cytotoxic T-Lymphocyte-Associated Protein 4
TIM-3	T-Cell Immunoglobulin and Mucin-Domain Containing-3
LAG-3	Lymphocyte Activation Gene-3
NK	Natural-killer cell
Treg	Regulatory T-cell
SMD	Standardized Mean Difference
AIC	Akaike Information Criterion
RSEM	RNA-Seq by Expectation-Maximization
IHC	Immunohistochemistry
HPA	Human Protein Atlas
