# Supplementary material for: SDK1 as an Independent Prognostic Biomarker in Primary Glioma: A Multi-Cohort Validation Study with Functional Characterization
Source: Int J Mol Sci. 2026 May 8;27(10):4199. doi: 10.3390/ijms27104199 (PMC13207070; doi:10.3390/ijms27104199)
Supplement: Supplementary file 1 [file ijms-27-04199-s001.zip › ijms-4228417-supplementary.pdf]

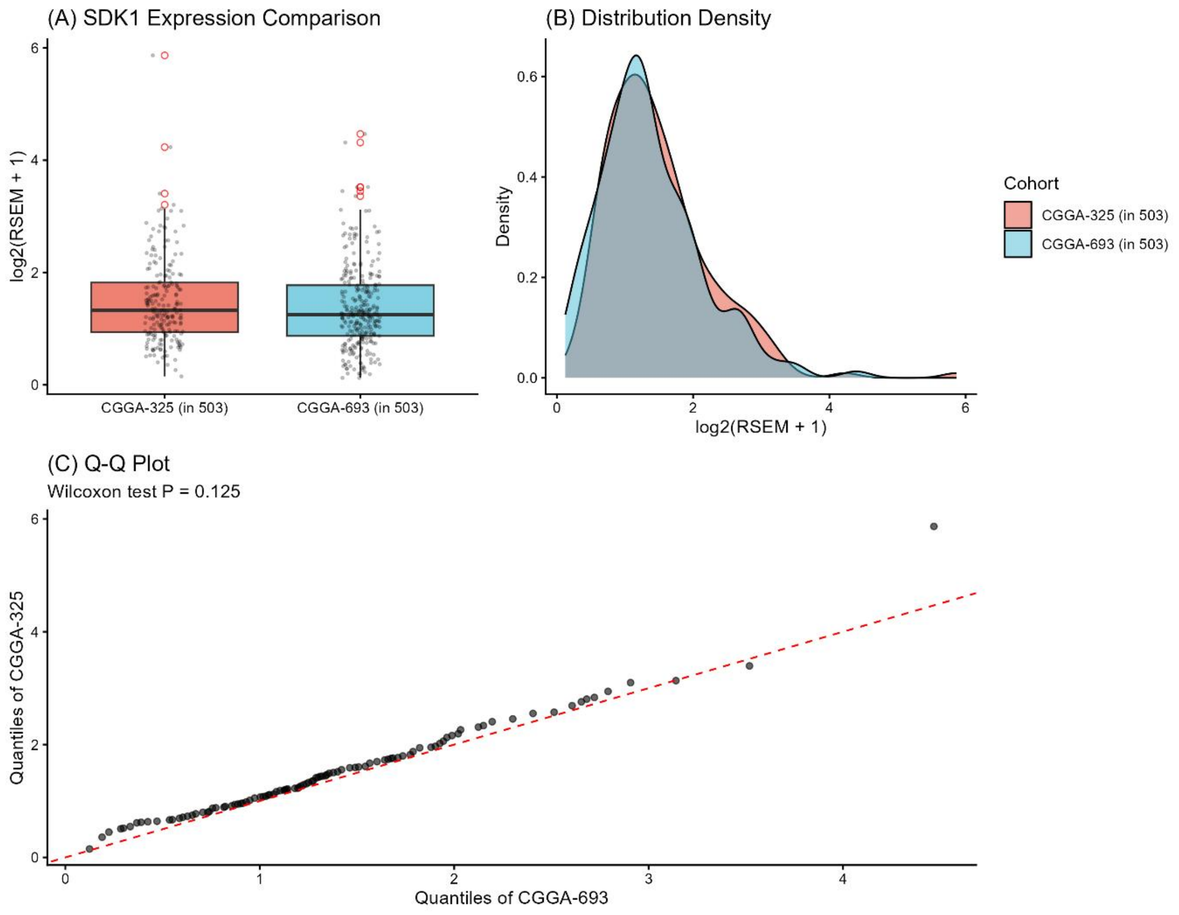

Supplementary Figure S1. Data pooling justification for CGGA sub-cohorts.

(A) Boxplot comparing SDK1 expression between CGGA-325 and CGGA-693 sub-cohorts.

(B) Density plots showing SDK1 expression distributions in each sub-cohort.

(C) Quantile–quantile plot demonstrating comparable distributional patterns between sub-cohorts. Wilcoxon rank-sum test P=0.125, supporting cohort pooling.

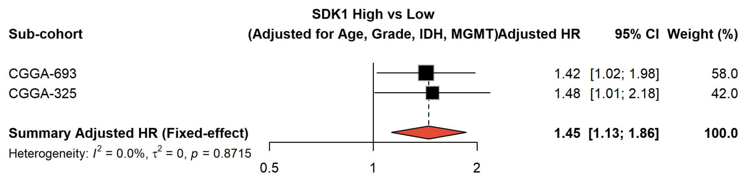

Supplementary Figure S2. Internal meta-analysis of SDK1 prognostic effect across CGGA sub-cohorts. For est plot showing hazard ratios for SDK1 in the CGGA-325 and CGGA-693 cohorts. Fixed-effect meta-anal ysis yielded a pooled HR of 1.45 (95% CI 1.13–1.86), with no heterogeneity observed between sub-cohort

s (I^2 = 0%).

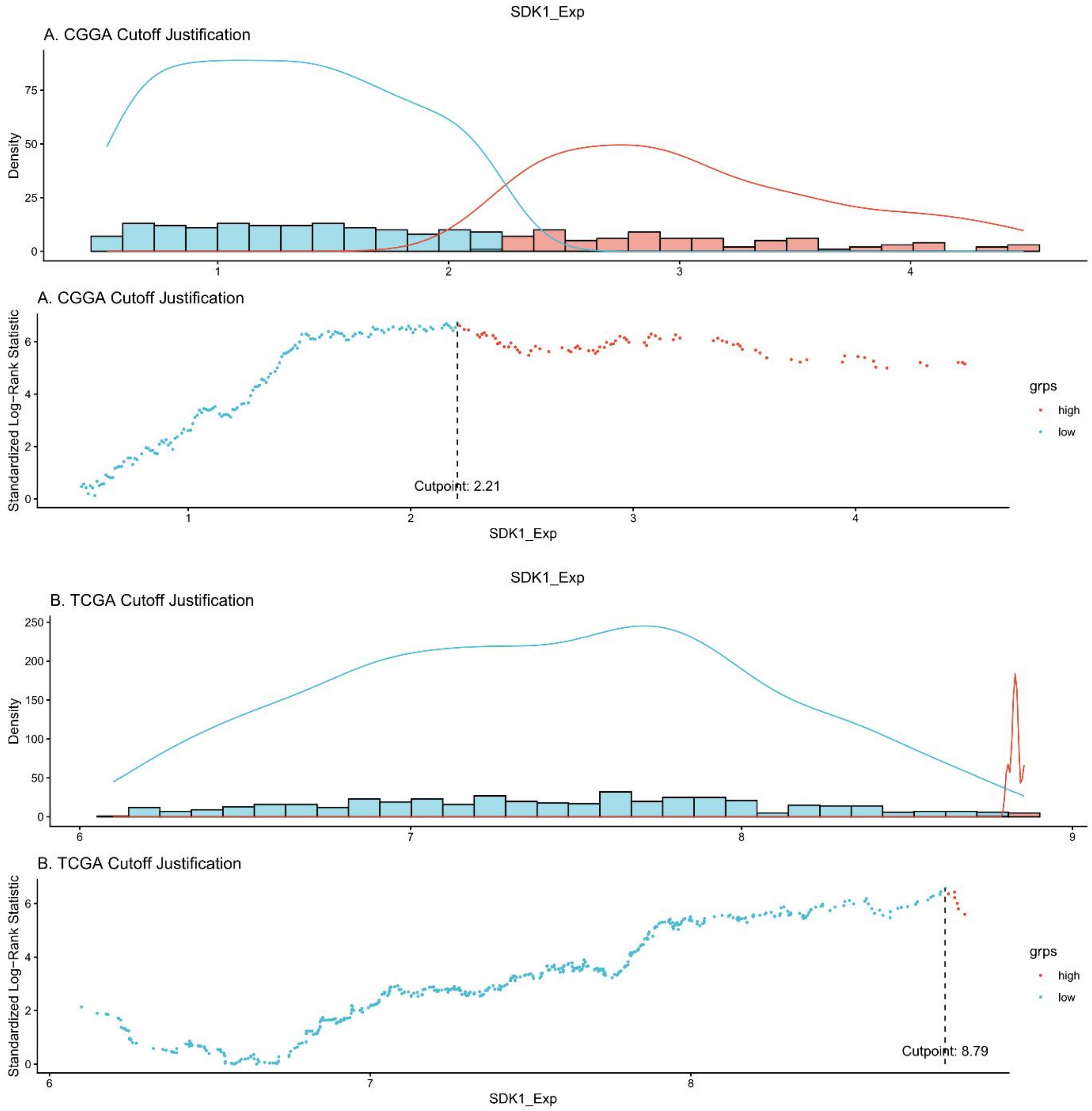

Supplementary Figure S3. Optimal cutoff determination using maximally selected rank statistics(maxstat). SDK1 expression cutoffs were determined independently for CGGA and TCGA cohorts using maximally selected rank statistics.

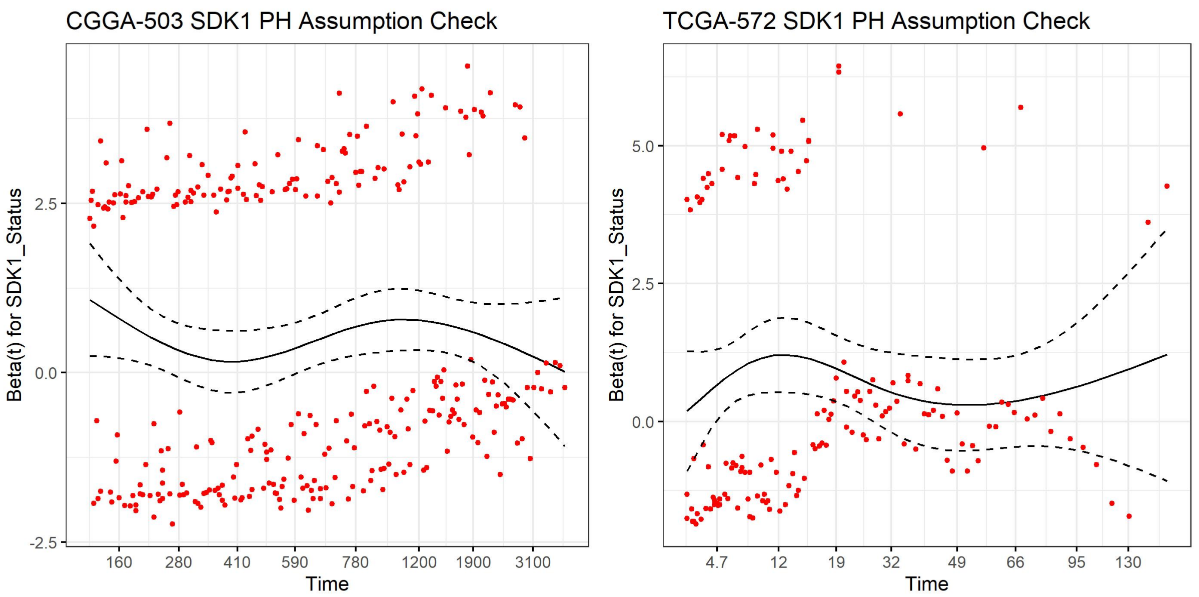

Supplementary Figure S4. Proportional hazards assumption assessment for SDK1. Schoenfeld residual plo t demonstrating that the proportional hazards assumption was satisfied for SDK1 in the Cox proportional hazards model.

(A)

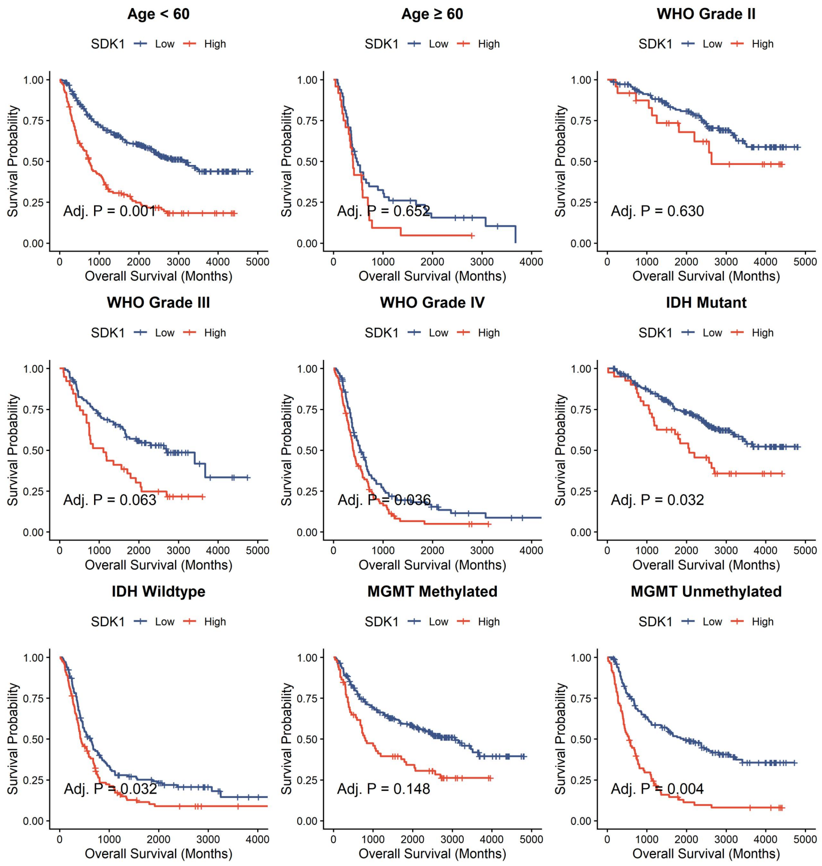

(B)

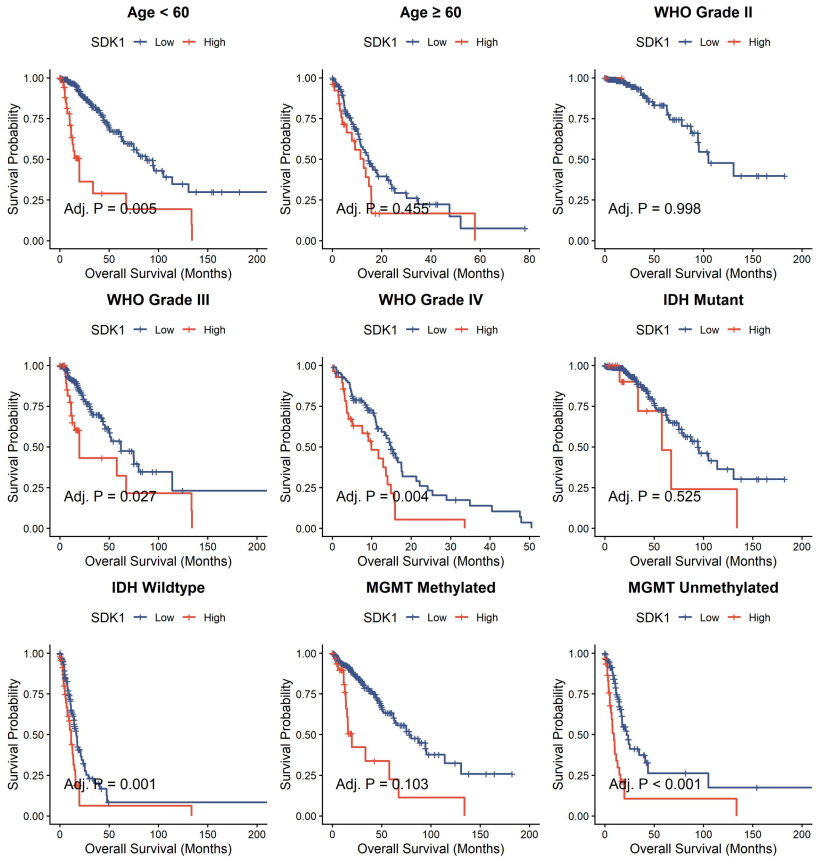

Supplementary Figure S5. Subgroup Kaplan–Meier survival curves in the CGGA cohort (A), TCGA cohor t(B).

Overall survival comparison between SDK1-high and SDK1-low groups stratified by age, WHO grade, ID H mutation status, and MGMT promoter methylation.

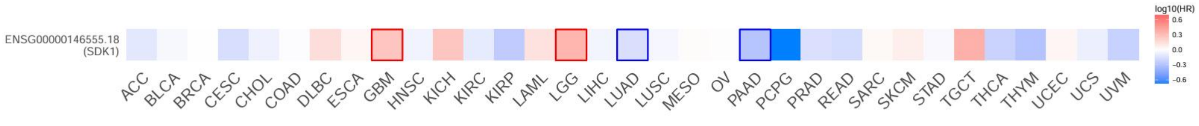

Supplementary Figure S6. Pan-cancer survival association of SDK1 expression. Heatmap showing the ass ociation between SDK1 expression and overall survival across TCGA cancer types, generated using the G EPIA2 platform. Colors represent log10 hazard ratios (HR) derived from Cox proportional hazards model s, with red indicating increased risk and blue indicating decreased risk associated with higher SDK1 exp ression. Notably, positive hazard ratios were observed in glioma cohorts (GBM and LGG), suggesting tha t elevated SDK1 expression is associated with poorer survival in glioma.

(A)

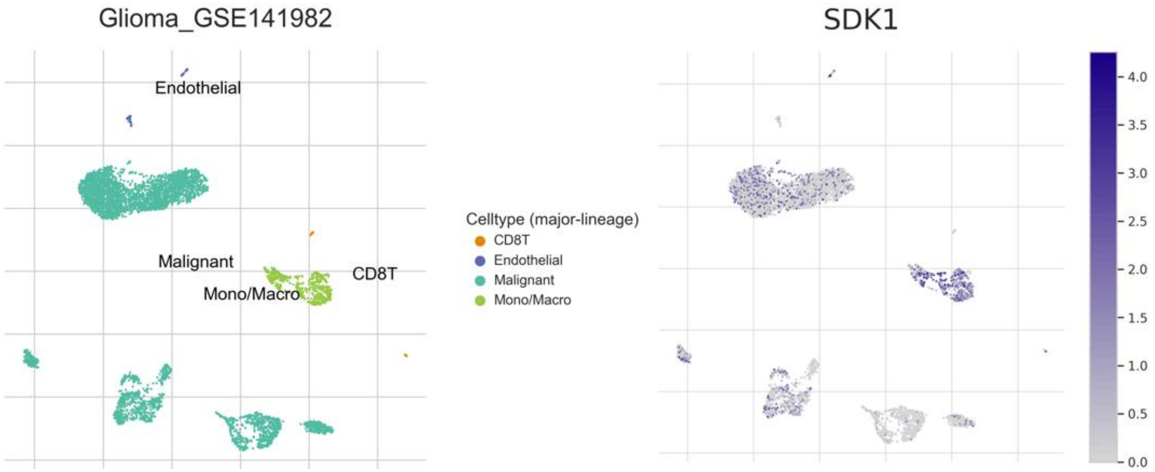

(B)

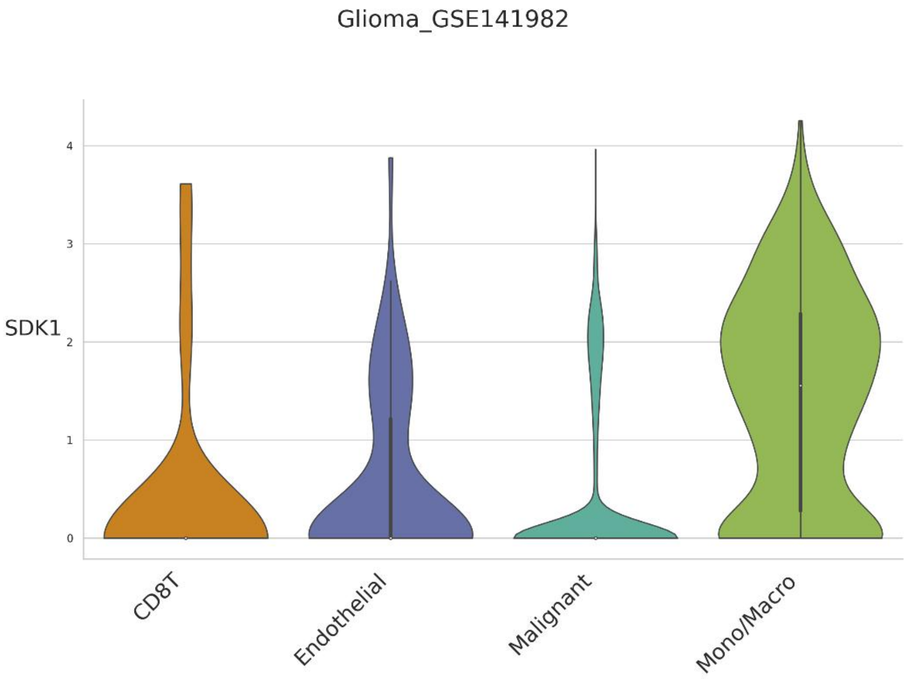

Supplementary Figure S7. Single cell RNA sequencing overview of SDK1 expression in glioma. (GSE1419
82). (A) UMAP visualization of the GSE141982 single-cell RNA-seq dataset showing major cell lineages (l
eft) and SDK1 expression levels (right). SDK1 expression is observed in subsets of malignant glioma cells
and endothelial cells and is most prominently enriched in monocyte/macrophage populations, while min
imal expression is detected in CD8 T cells. (B) Violin plot illustrating the distribution of SDK1 expressio
n across major cell lineages. SDK1 expression is highest in monocyte/macrophage cells, with detectable e
xpression in endothelial and malignant cell populations, whereas CD8 T cells show minimal expression.

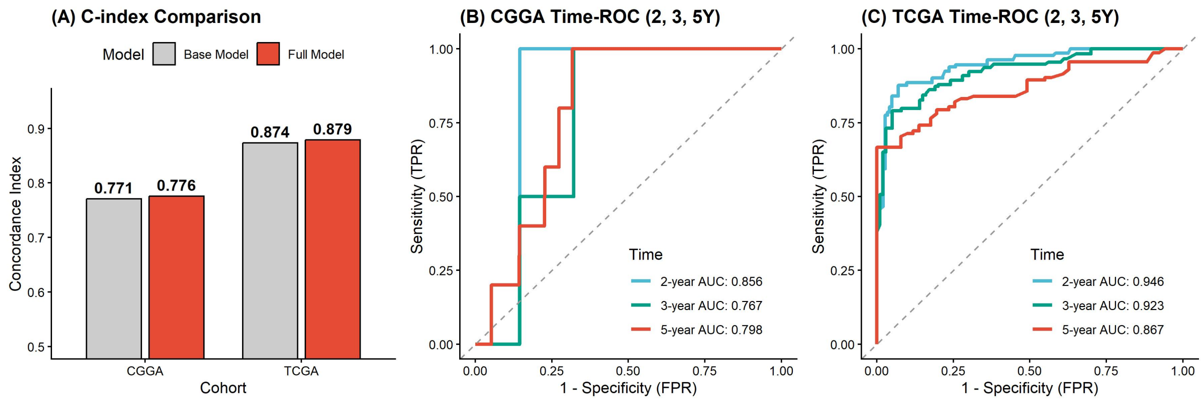

Supplementary Figure S8. Incremental predictive value of SDK1. (A) C index comparison between the base model (age, WHO grade, IDH status, MGMT methylation) and the full model additionally including SDK1 status in the CGGA training cohort and the TCGA validation cohort. SDK1 addition yielded mod est C index improvements (CGGA: 0.771→0.776; TCGA: 0.874→0.879; Δ≈+0.005 in both cohorts). (B–C) Ti me dependent ROC curves of the full model at 2 , 3 , and 5 year timepoints in CGGA (B) and TCGA (C), showing acceptable discrimination in CGGA (AUCs 0.856, 0.767, 0.798) and excellent discrimination i n TCGA (AUCs 0.946, 0.923, 0.867).

* Time-dependent ROC curves were assessed at 2, 3, and 5 years because 1-year AUC estimation was no t available in one cohort.

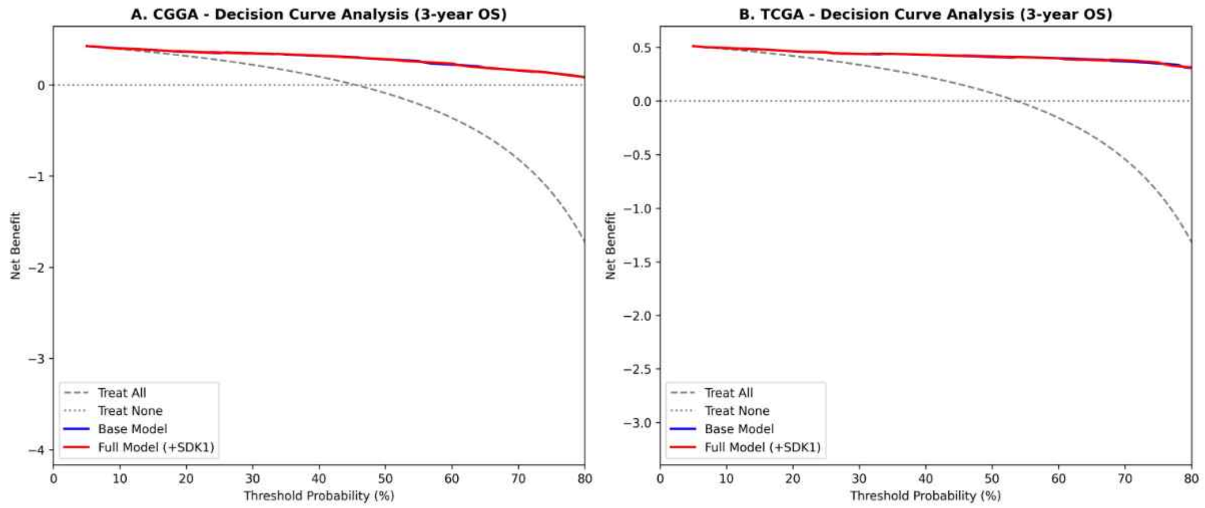

Supplementary Figure S9. Decision Curve Analysis (DCA) evaluating the clinical utility of the SDK1-inclusive prognostic model.

Net benefit curves for the base model (Age, WHO grade, IDH, MGMT) and the full model (+SDK1) are shown for 3-year overall survival in the CGGA and TCGA cohorts. The full model demonstrated a marginal net benefit across threshold probabilities of 24–87% (CGGA) and 33–87% (TCGA), suggesting that adding SDK1 expression status provides adjunctive value in clinical risk stratification beyond established markers.

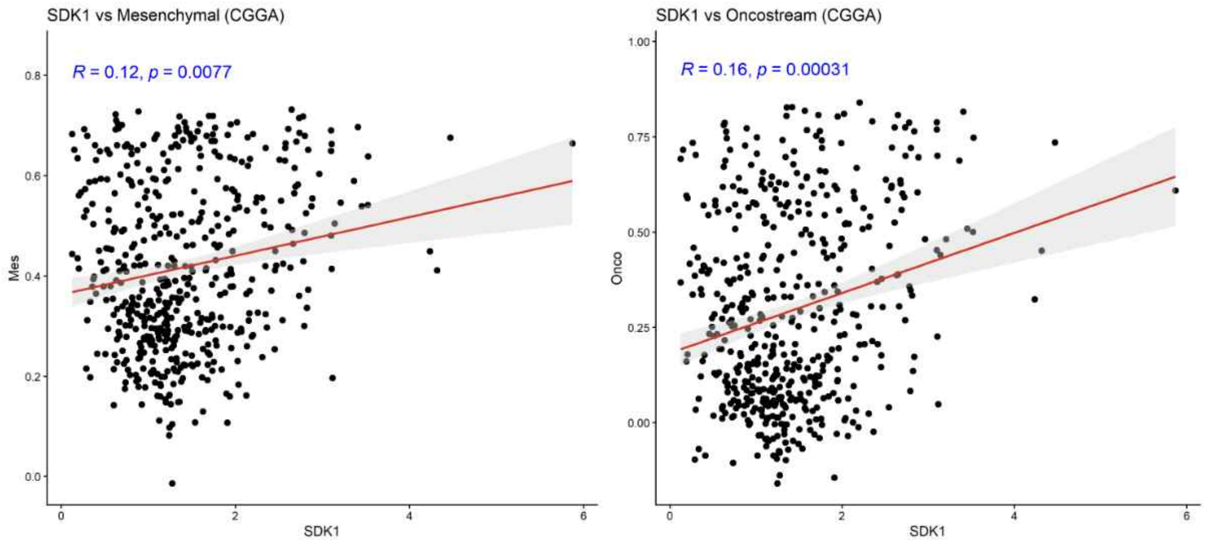

Supplementary Figure S10. Correlation between SDK1 expression and the COL1A1-associated mesenchymal program score.

Scatter plots showing Spearman correlation between SDK1 transcript levels and the mean z-score of the 10-gene mesenchymal program (COL1A1, COL1A2, COL3A1, COL5A1, FN1, VIM, ACTA2, TAGLN, POSTN, SPARC). A weak but statistically significant positive correlation was observed in both CGGA (R=0.12, P=0.008) and TCGA (R=0.15, P<0.001), supporting SDK1's peripheral positioning within the mesenchymal transcriptional landscape.

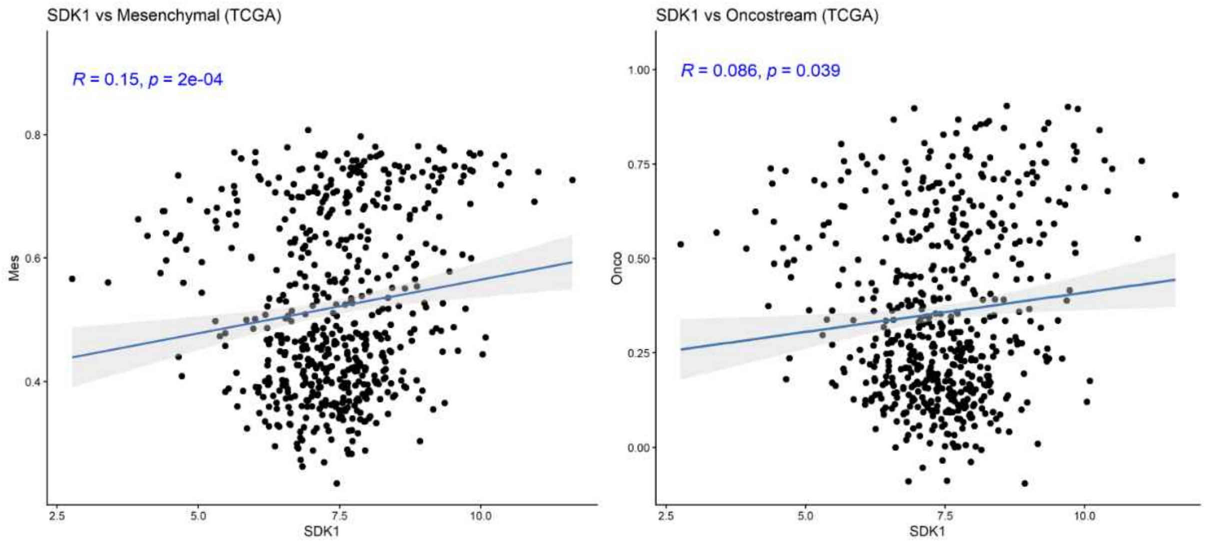

Supplementary Figure S11. Correlation between SDK1 expression and the oncostream-related gene signature.

Spearman correlation analysis with the oncostream signature score (SERPINE1, THBS1, TAGLN, LGALS1, ANXA2, S100A11, MYL9). SDK1 expression showed weak, positive associations in both cohorts (CGGA: R=0.16, P<0.001; TCGA: R=0.086, P=0.039), linking SDK1 to programs mediating spatially organized collective invasion in glioblastoma.

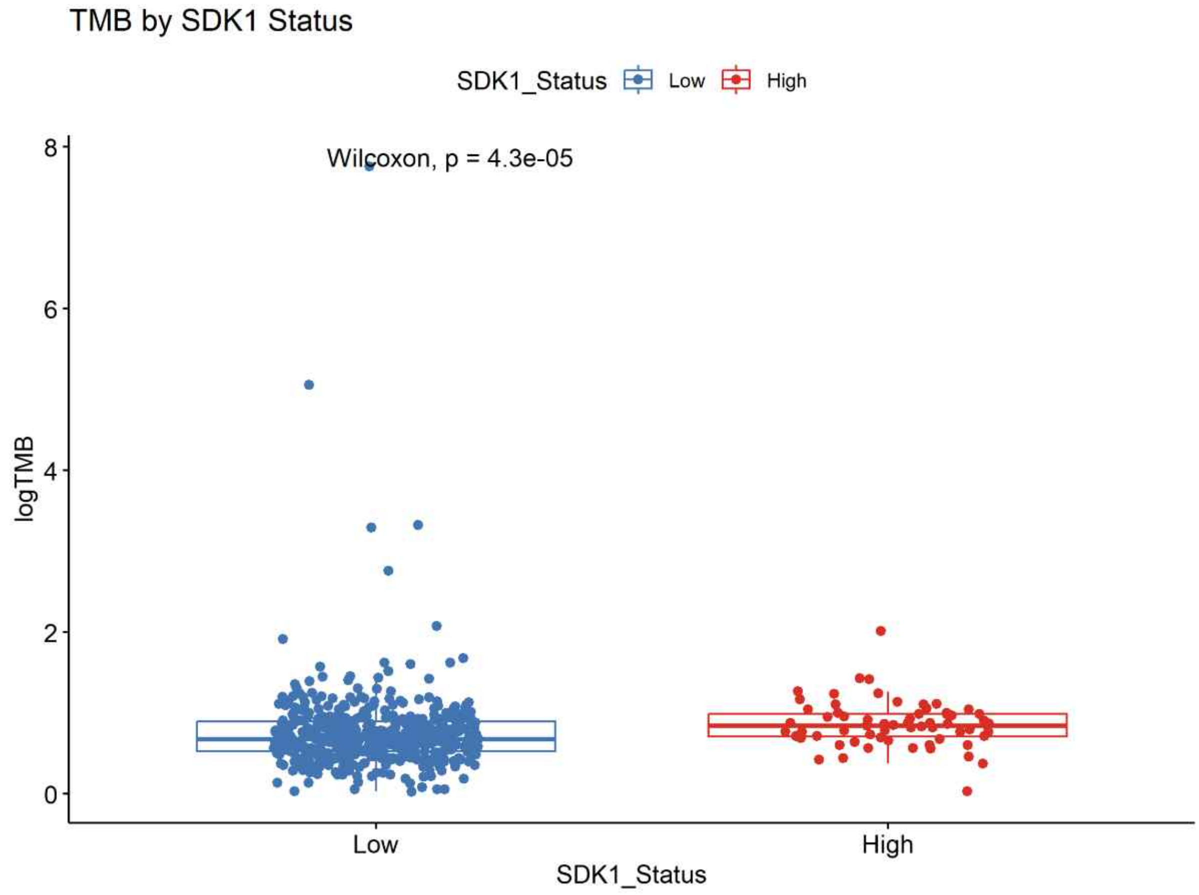

Supplementary Figure S12. Comparison of tumor mutational burden (TMB) by SDK1 expression

status.

Box plots comparing the total number of non-synonymous somatic mutations between SDK1-high and SDK1-low groups in the TCGA cohort. While SDK1-high tumors showed a significantly higher TMB (Wilcoxon $P=4.3 \times 10^{-5}$), the absolute difference in median values was modest. Continuous SDK1 expression did not correlate significantly with TMB, and no interaction was found in survival analysis, suggesting SDK1's effect is primarily microenvironment-driven .

Supplementary Table S1. CGGA-325 vs. CGGA-693 clinical characteristics.

Characteristics	Level	CGGA-325	CGGA-693	P-value
**n**		207	296	
**Age (mean (SD))**		44.51 (12.27)	44.68 (13.14)	0.883
**Age_Group (%)**	<60	180 (87.0)	251 (84.8)	0.582
	>=60	27 (13.0)	45 (15.2)	
**Grade (%)**	WHO II	81 (39.1)	85 (28.7)	0.003
	WHO III	43 (20.8)	100 (33.8)	
	WHO IV	83 (40.1)	111 (37.5)	
**IDH (%)**	Mutant	102 (49.3)	156 (52.7)	0.505
	Wildtype	105 (50.7)	140 (47.3)	
**MGMT (%)**	Methylated	95 (45.9)	171 (57.8)	0.011
	Un-methylated	112 (54.1)	125 (42.2)	
**SDK1_Status (%)**	Low	139 (67.1)	213 (72.0)	0.289
	High	68 (32.9)	83 (28.0)	

Supplementary Table S2. Proportional hazards assumption check.

Cohort	Variable	Chi-Square	df	P-value	Status
**CGGA Cohort**	Age	1.55	1	0.213	Satisfied
	Grade	10.06	2	0.007	Violated
	IDH	20.85	1	< 0.001	Violated
	MGMT	2.16	1	0.142	Satisfied
	SDK1 Status	0.17	1	0.680	Satisfied
	GLOBAL (Overall Model)	26.49	6	< 0.001	Violated
**TCGA Cohort**	Age	2.79	1	0.095	Satisfied
	Grade	3.83	2	0.148	Satisfied
	IDH	11.29	1	< 0.001	Violated
	MGMT	4.16	1	0.041	Violated
	SDK1 Status	0.27	1	0.603	Satisfied
	GLOBAL (Overall Model)	21.49	6	0.001	Violated

Supplementary Table S3. Sensitivity analysis: primary vs. stratified Cox.

Cohort	Model	Stratified Variables	SDK1 HR (95% CI)	P-value
**CGGA Cohort**	Primary Model	Grade, IDH	1.46 (1.14 - 1.86)	0.002
	Stratified Model	Grade, IDH	1.40 (1.10 - 1.79)	0.007
**TCGA Cohort**	Primary Model	IDH, MGMT	1.98 (1.34 - 2.93)	< 0.001
	Stratified Model	IDH, MGMT	1.97 (1.33 - 2.94)	< 0.001

Supplementary Table S4. Comparison of baseline characteristics between included and excluded patients due to missing data.

Characteristics	Level	Excluded	Included	P-value
**n**		515	503	
**Age (mean (SD))**		41.77 (11.55)	44.61 (12.77)	<0.001
**Grade (%)**	Grade II	125 (24.5)	166 (33.0)	0.002
	Grade III	191 (37.5)	143 (28.4)	
	Grade IV	194 (38.0)	194 (38.6)	
**IDH (%)**	Mutant	273 (59.0)	258 (51.3)	0.020
	Wildtype	190 (41.0)	245 (48.7)	
**MGMT (%)**	Methylated	206 (59.7)	266 (52.9)	0.058

	Unmethylated	139 (40.3)	237 (47.1)	

Characteristics	Level	Excluded	Included	P-value
**n**		550	572	
**Age (mean (SD))**		57.02 (14.48)	46.77 (15.30)	<0.001
**Grade (%)**	Grade II	2 (0.4)	214 (37.4)	<0.001
	Grade III	0 (0.0)	241 (42.1)	
	Grade IV	473 (99.6)	117 (20.5)	
**IDH (%)**	Mutant	80 (18.9)	377 (65.9)	<0.001
	Wildtype	343 (81.1)	195 (34.1)	
**MGMT (%)**	Methylated	188 (52.2)	425 (74.3)	<0.001
	Unmethylated	172 (47.8)	147 (25.7)	

Supplementary Table S5. Net Reclassification Improvement (NRI) analysis comparing the base model (Age, WHO Grade, IDH, MGMT) and the full model (Base + SDK1) at 3-year overall survival. Risk categories: Low (<20%), Medium (20–50%), High (>50%). Bootstrap 95% CIs based on 1,000 iterations.

**Sample**	Events / Non-events	216 / 258	116 / 100
**Events**	Reclassified UP	7 (3.2%)	1 (0.9%)
	Reclassified DOWN	0 (0.0%)	1 (0.9%)
	Unchanged	209 (96.8%)	114 (98.3%)
	NRI (events)	0.0324	0.0000
**Non-events**	Reclassified UP	14 (5.4%)	0 (0.0%)
	Reclassified DOWN	0 (0.0%)	1 (1.0%)
	Unchanged	244 (94.6%)	99 (99.0%)
	NRI (non-events)	−0.0543	0.0100
**Categorical NRI**	Estimate (95% CI)	−0.02 (−0.06 to 0.01)	0.01 (−0.02 to 0.05)
	P-value	1.000	0.566
**Continuous NRI**	Estimate (95% CI)	0.50 (0.34 to 0.68)	0.50 (0.29 to 0.69)
	P-value	<0.001	<0.001
**Discrimination**	Base model AUC	0.879	0.936
	Full model AUC	0.884	0.937
	ΔAUC	+0.005	+0.002

Risk categories for categorical NRI: Low (<20%), Medium (20–50%), High (>50%). Continuous NRI assesses reclassification without predefined risk categories. Bootstrap 95% CIs and P-values based on 1,000 iterations. AUC = area under the receiver operating characteristic curve.

Supplementary Table S6. Partial correlation analysis of SDK1 expression with immune cell infiltration signatures, controlling for the COL1A1-associated mesenchymal program score. Original correlations (unadjusted Spearman) and partial correlations (mesenchymal-adjusted Spearman) are shown for each immune cell type across both cohorts.

CGGA Cohort (N=503)

**M2 Macrophage**	**0.147**	**9.72×10^{-4}**	**0.093**	**0.038**	**↓ Reduced**
**Treg**	0.053	0.236	−0.027	0.539	NS → NS
**CD8+ T cell**	**0.226**	**2.94×10^{-7}**	**0.199**	**7.32×10^{-6}**	**→ Maintained**
**CD4+ T cell**	**0.143**	**1.26×10^{-3}**	**0.091**	**0.043**	**↓ Reduced**
**NK cell**	**0.174**	**8.60×10^{-5}**	**0.162**	**2.56×10^{-4}**	**→ Maintained**
**B cell**	**0.223**	**4.54×10^{-7}**	**0.194**	**1.42×10^{-5}**	**→ Maintained**
**Dendritic cell**	**0.175**	**7.87×10^{-5}**	**0.131**	**3.21×10^{-3}**	**↓ Reduced**

TCGA Cohort (N=572)

**M2 Macrophage**	**0.241**	**5.49×10^{-9}**	**0.188**	**6.30×10^{-6}**	**↓ Reduced**
**Treg**	−0.039	0.347	**−0.158**	**1.49×10^{-4}**	**NS → Sig***
**CD8+ T cell**	−0.039	0.355	**−0.084**	**0.044**	**NS → Sig***
**CD4+ T cell**	**0.241**	**5.36×10^{-9}**	**0.193**	**3.47×10^{-6}**	**→ Maintained**
**NK cell**	−0.011	0.798	**−0.107**	**0.011**	**NS → Sig***
**B cell**	−0.003	0.939	**−0.089**	**0.034**	**NS → Sig***
**Dendritic cell**	**0.194**	**3.04×10^{-6}**	**0.129**	**1.93×10^{-3}**	**↓ Reduced**

→ Maintained = partial rho ≥80% of original; **↓ Reduced** = 50–80% of original; **↓↓ Attenuated** = <50% of original; **Lost** = significant → non-significant; **NS → Sig*** = originally non-significant but became significant after adjustment (suppressor effect).

Green-shaded cells indicate statistical significance (P<0.05). Partial correlations control for the COL1A1-associated mesenchymal program score (mean z-scored expression of COL1A1, COL1A2, COL3A1, COL5A1, FN1, VIM, ACTA2, TAGLN, POSTN, SPARC).

Supplementary Table S7. Multivariate Cox Regression Analysis Including SDK1 and TMB Interaction

Cohort: TCGA (n = 569, Events = 150)

Variable	Coefficient	HR	95% CI (Lower-Upper)	P-value
**Age Group (≥60 vs. <60)**	0.944	2.571	1.748 – 3.782	< 0.001*
**Grade (G3 vs. G2)**	0.729	2.073	1.209 – 3.554	0.008*
**Grade (G4 vs. G2)**	1.487	4.423	2.312 – 8.460	< 0.001*
**IDH Status (WT vs. Mut)**	1.690	5.422	3.043 – 9.658	< 0.001*
**MGMT (Unmethylated vs. Meth)**	0.071	1.073	0.733 – 1.572	0.716
**Log2(TMB + 1)**	-1.130	0.323	0.117 – 0.894	0.030*
**SDK1 Status (Low vs. High)**	-1.217	0.296	0.107 – 0.820	0.019*
**Log2(TMB + 1) × SDK1 (Low)**	0.802	2.230	0.792 – 6.282	0.129
